# Double Network as a Design Paradigm for Structuring Emulsion Gels in Food

**DOI:** 10.1111/1541-4337.70201

**Published:** 2025-05-15

**Authors:** Canice Chun‐Yin Yiu, Yong Wang, Cordelia Selomulya

**Affiliations:** ^1^ School of Chemical Engineering UNSW Sydney NSW Australia

## Abstract

Emulsion gels can be potentially used to structure lipids when developing novel plant‐based food products. Current emulsion gels comprising a single polymeric network in the continuous phase are limited in their ability to deliver the desired textural and functional properties. In biomedical engineering, double‐network hydrogels are extensively used. Here, the concept was applied to create double‐network emulsion gels with enhanced stability, texture, and encapsulation of bioactive ingredients to closely mimic animal‐based food. The existence of a second network is crucial to tuning the thermal characteristics for cooking stability and controlled release for functional food applications. The textural and thermal characteristics in dual gel network systems could be modulated by varying the concentration of individual biopolymers and/or gelators. Albeit the improvement compared to a single gel system, there are still challenges in creating double‐network emulsion gel systems from food proteins and polysaccharides, mainly due to the differences in osmotic pressure of the hydrophilic continuous polysaccharide gel network and the hydrophobic dispersed oil phase. This led to apparent phase separations in these mixed protein‐polysaccharide systems that have negative implications on the gel strength of these gels. This review provides a summary of the current understanding of double‐network emulsion gels in terms of formation, network interaction, and implications on their properties relevant to food processing and product applications. The double‐network emulsion gels could be a better option for structuring lipids than single‐network emulsion gels and oleogels in plant‐based food products.

## 1 Introduction

The versatile use of gels encompasses various applications, from carriers of bioactive ingredients to the creation of food analogs. Single network emulsion gels are soft materials that consist of stabilized oil droplets, encased and immobilized by a three‐dimensional polymeric network in the continuous aqueous phase. For food applications, the continuous phase typically consists of proteins and polysaccharides interacting through physical and/or chemical interactions. Likewise, the amphiphilicity of protein and certain polysaccharides is responsible for the stabilization of the dispersed phase. Emulsion gels have garnered interest due to their structural similarity to animal tissues and animal‐derived foods. Like the case for adipose tissue, where lipocytes are immobilized in a matrix of connective tissues, or cheese, where milk fat is encased within a casein network (Ren et al. 2022). Compared with other lipid structuring regimes, such as oleogels, emulsion gels typically require lower gelation temperatures during their formation, thus preventing the degradation of bioactive ingredients or oxidation of lipids (Lin et al. 2020; Valle et al. 2024; Zetzl et al. 2012). Food categories such as dairy, confectionery, and condiments are typical emulsion‐based foods encountered in our diets. As they contain both lipid and aqueous phases, emulsion gels have been investigated as controlled‐release delivery devices for hydrophilic and lipophilic bioactive ingredients (Yiu et al. 2023).

On the other hand, double‐network hydrogels are characterized by the existence of two interpenetrating polymeric networks within a three‐dimensional aqueous phase. These gels have been adopted in biomedical and tissue engineering for creating materials with enhanced mechanical strength and increased functionality, as the advantages of each network could be complementary to the target application (Gu et al. 2018; X. Xu et al. 2021). Efforts to apply the double network approach to create highly texturized food hydrogels were observed recently to mimic the tough texture of beef reticulum and rumen (Du et al. 2023).

Most studies to date remain focused on emulsion gels with a single network as their structural formation. As the demand for novel food rises due to global challenges in human health and the environment, a prominent need exists to develop more versatile food gels with targeted properties. Challenges persist in tailoring properties such as stability, texture, and other functional properties with the current emulsion gel frameworks. In single‐network emulsion gels, the delivery of these properties is shouldered by the single biopolymer. Such that, in cases where the strength of the network is not sufficient, another solid material was used. For instance, saturated fat was used to increase gel firmness (Dreher et al. 2020; Oliver et al. 2015). Thus, emulsion gels with complementing polymeric networks could be a possible solution to improve texture with better health implications without using saturated fat. Moreover, despite single‐network emulsion gels being successful in the protection of bioactive ingredients and probiotics in both phases, a recent study showed that the inclusion of a second polymeric network could offer better protection and modulation of release in double‐network emulsion gels. (Ghiraldi et al. 2021; Lu et al. 2019; Qin et al. 2023). Therefore, a double‐network schematic could enable the creation of healthier texturized food with greater control over its performance for improved effectiveness in delivering desirable characteristics.

This review seeks to outline the current progress in the development of double‐network emulsion gels in relation to network interactions and their implications on gel properties. From these properties, potential applications of double‐network emulsion gels in food were examined to demonstrate the benefits of using double‐network gels in new products.

## 2 Double‐Network Gels in Food

### 2.1 Double‐Network Hydrogels: Network Formation and Interaction

The existence of two interpenetrating or semi‐interpenetrating networks in double‐network hydrogel allows for the mechanical strength of the gel to be enhanced (Yin et al. 2023). The intra‐ and internetwork interactions may be guided by covalent and/or physical interactions like hydrogen bonds, hydrophobic interactions, and electrostatic interactions (Yin et al. 2023; Zhang et al. 2019). In food, double‐network hydrogels focus on the use of biopolymers as the building block for the two networks. These hydrogels could function as edible delivery systems, as well as plant‐based meat mimetics, owing to their enhanced resistance to environmental stressors and mechanical strength (Yin et al. 2023).

The biopolymers that could be used in these hydrogels include both naturally extracted proteins and polysaccharides, as well as modified variants of biopolymers. Double‐network hydrogels have been created using double protein, double polysaccharide, and mixed protein‐polysaccharide systems (Yin et al. 2023). Common protein candidates in double‐network food gels include whey protein, gelatin, and pea protein. Polysaccharides used in double‐network food gels are alginates, carrageenan, cellulose, konjac glucomannan (KGM), gellan gum, and starches. The gelation of these biopolymers can be done through the addition of food‐grade gelators like salts, acids, and enzymes, or processing means such as heat or shear (McClements 2024).

The networks could form sequentially (two‐step) or simultaneously (one‐step), where the gel‐inducing process may be common or varying between proteins and polysaccharides. A two‐step process, in which each network was formed via its respective gelling condition, offers greater control over the gel formation as each network could be tailored and adapted as needed. A two‐step process may be significantly slower than a one‐step process, as second network formation requires additional processing, handling, and/or incubation (Chen et al. 2015; Du et al. 2021). These limitations could be partially remedied through a “one‐pot” method, where both monomers are dissolved in the initial solution and gelled by varying processing conditions (Chen et al. 2015; Du et al. 2021; Du et al. 2022). On the other hand, a one‐step process allows for the rapid formation of a double‐network system with increased uniformity (Li et al. 2016). This is achieved by the co‐addition of gelators or the use of a common processing pathway. Nonetheless, protein and polysaccharides share similar gel‐initiation, despite differences in their chemical structure (Du et al. 2021; Kim et al. 2024). A range of factors, such as the type and concentration of gelators, thermal processing, and the relative concentration and molecular weight of each biopolymer, may affect the inter and intramolecular interactions within the gel microstructure. As such, the design of food double‐network gels needs to account for these factors during fabrication to optimize the desired characteristics (Du et al. 2021).

In a protein‐polysaccharide mixed system, where calcium ions may gel both pea protein and sodium alginate (SA), Wang et al. (2022) described a two‐step, one‐pot method to create a double‐network hybrid interpenetrating gel. The first network, which covalently crosslinks pea protein with TGase, was formed before submerging the gel in a calcium chloride solution for 18 h to create the second network. Conversely, a one‐step method is sufficient to create a physical double‐network gel between protein and polysaccharide with interactions between the two networks. Du et al. (2022) described a gelatin/agarose gel created simply by sequential heating and cooling. ATR‐FTIR analysis showed that the interactions between the networks were through hydrogen bonds, with no covalent bonds formed between the two species (Du et al. 2022). Furthermore, in protein‐polysaccharide gels created by heat and oxidase treatment, the degree of interpenetration and linked interaction between gels may also be modulated by the protein/polysaccharide ratio and enzyme concentration (Chen et al. 2019).

On the other hand, an interpenetrating double‐network hydrogel may also be formed in a one‐pot, one‐step method using both charged and neutral polysaccharides. Amici et al. (2002) described a κC and agar double‐network gel formed by exploiting the charged nature of κC. Although both agar and κC may be gelled by sequential heating and cooling, the addition of potassium salt into the mixture selectively binds with the carrageenan to elevate its gelation temperature and promotes carrageenan–carrageenan interactions. As shown by Tao et al. (2022), excessive addition of ions may disrupt the formation of the double helix of high‐acyl gellan gum (HAG) in a double‐network gel with agarose, which results in a weakened gel. This may be rectified by immersing the cooled gel in calcium chloride rather than the addition of salt prior to cooling. The immersion of a set gel after the gelation of a first network is preferred if the second network requires additional gelators or modification. Such was the case for the creation of a DKG/agarose gel, where a set gel of agarose and KGM was first created before the gel was immersed in sodium carbonate for the deacetylation of KGM (Du et al. 2023).

A different prospect exists when attempting to form an interpenetrating network of two proteins, where the selection of proteins is critical to forming a double‐network gel. In mixed‐protein systems between whey and soy, the one‐step heat‐induced gelation created a singular network as whey and β‐conglycinin share a similar denaturation temperature. The singular network was observed to have a storage modulus (G’) that is between the pure gel of the respective species at a constant total protein content. However, the composite gel is stronger than the sum of the individual gels at their respective ratio (Jose et al. 2016). However, an interpenetrating network may be formed where the two proteins have different denaturation temperatures. Such a case was observed as demonstrated by Wu et al. (2019) for cod actin (73–74°C) and soy protein (77°C and 94°C). Moreover, double‐network protein gels may also be formed by two proteins that gel via contrasting interactions that are affected by their native amino acid composition. Interpenetrating protein networks were able to be formed from a prolamin and a globulin. Globulin, like soy protein, is limited in free sulfhydryl groups that would not readily form disulfide bonds with cystine‐rich prolamin, such as gluten, during heating (Cornet et al. 2020; Lambrecht et al. 2017). Moreover, the lack of Lys in zein protein, and its low water solubility were not found to have formed co‐protein with soy/pea in the presence of TGase (Mattice and Marangoni 2021). Therefore, it is apparent that success in making double‐network protein gel lies within the correct pairing of proteins.

### 2.2 Design of Double‐Network Emulsion Gels in Food

Double‐network emulsion gels add a further layer of complexity compared with double‐network hydrogel, as it is crucial to maintain emulsion stability and understand the role of the dispersed phase in the network.

Both proteins and polysaccharides may be used as emulsifiers to stabilize the oil/water (O/W) interface. Proteins are amphiphilic species that contain both hydrophobic and hydrophilic amino acids, allowing them to adsorb at the surface of oil droplets (Yiu et al. 2023). In contrast, most polysaccharide chains are hydrophilic, resulting in poor emulsifying properties (Nakauma et al. 2008). Certain polysaccharides, such as sugar beet pectin (SBP) and gum arabic, contain hydrophobic moieties on the chain, allowing them to stabilize the O/W interface. The viscosity‐inducing, along with their steric and electrostatic effects, hinder the flocculation and aggregation of droplets, which result in the stability of the emulsion (Miao et al. 2021; Nakauma et al. 2008). Furthermore, Pickering emulsions may be formed by micro‐ or nanoparticles of protein and polysaccharides. Pickering particles are spherical solids that adsorb at the O/W interface. The partial wetting of the particle by both fluids creates a physical and an energy barrier against coalescence and Ostwald ripening (Sarkar and Dickinson 2020; Schroen et al. 2024). In food, Pickering emulsions, mixed interfaces were identified to be more common in food‐based Pickering particles due to the existence of surface‐active components (Schroen et al. 2024). The role of various surfactant types and Pickering particles is indispensable as the stability at the interface is key to the quality of the emulsion gel product.

**FIGURE 1:**
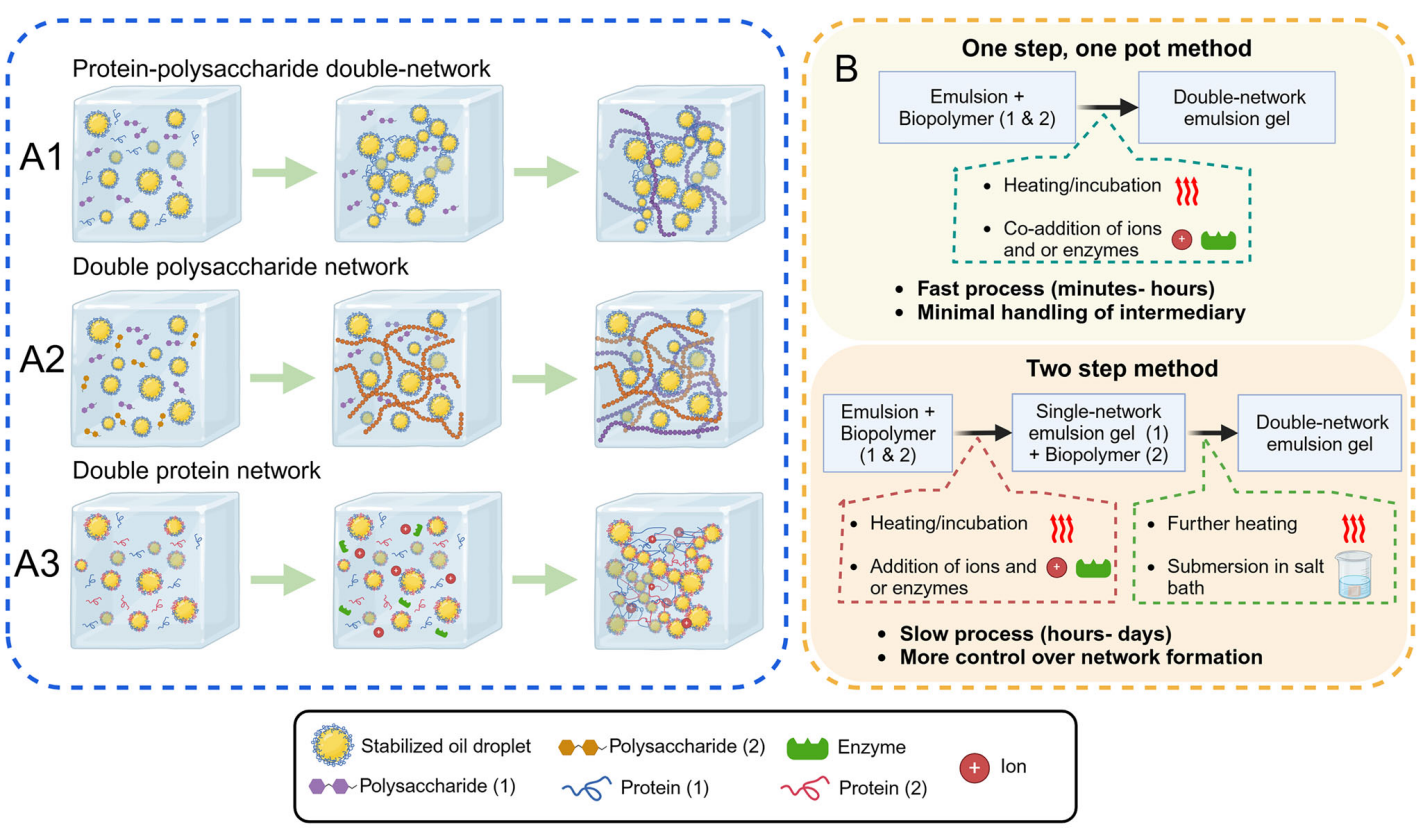
Schematic illustration of double‐network emulsion gels and their formation process. (a) Graphical illustration of the progression of gelation from an emulsion with constituent biopolymers (leftmost) to a double‐network emulsion gel (rightmost). (a1) Double‐network formed by proteins and polysaccharides showing an interpenetrating polysaccharide network with a protein‐stabilized and crosslinked oil droplet network. (a2) Double‐network formed by interpenetrating polysaccharide networks with a dispersed oil phase. (a3) Double‐network formed and stabilized by proteins with varying methods of cross‐linking. (b) Typical formation mechanisms of double‐network emulsion gels with their advantages and disadvantages.

In addition, the dispersed droplet may act as an integral part (active filler) or a noninteracting component (inactive filler) within an emulsion‐filled gel, which could impact its mechanical properties (Dickinson 2012). In emulsion gels where the dispersed phase participates as an active filler, gel strengthening was typically observed. As an active filler, the emulsion gel would also be more akin to animal tissues, where cells adhere to the connective network (van Oosten et al. 2019). In the formation of a double‐network emulsion gel, the overlapping of the emulsifying and structuring biopolymer was seen to strengthen the participation of the dispersed phase in the gel network. This is illustrated in Figures 1a1,a3, as protein‐stabilized oil droplets could form an interpenetrating network with other protein or polysaccharide molecules. On the other hand, the hydrophilicity of polysaccharides was highlighted in Figure 1a2 as a typical emulsion‐filled gel where amphiphilic species‐stabilized droplets are dispersed within interpenetrating polysaccharide networks. The above factors highlight the importance of the combination and compatibility between biopolymers in double‐network emulsion gel, as another degree of freedom was introduced beyond single‐network systems.

The formation mechanism of double‐network emulsion gel systems could be imprinted from their hydrogel counterparts, as the network largely exists in the hydrophilic phase. Both two‐step and one‐step formation mechanisms exist for a double‐network emulsion gel. Figure 1b outlines the common formation mechanism of double‐network emulsion gels in food. The gelation process usually begins with blending biopolymers and gelators into a stock emulsion stabilized by one of the biopolymers or a small molecule emulsifier (SME). The process then could be accompanied by heat processes to initiate gelation or dispersion for cold‐set gelation. Like double‐network hydrogels, two‐step processes were identified using different gelation conditions that were tailored for respective biopolymers. This could manifest as changing heating conditions or the submersion of the intermediate emulsion gel into a salt bath to initiate the gelation of the second network.

The added complexity, albeit with existing similarities to existing single‐network emulsion gels and double‐network hydrogels, creates great opportunities for developing double‐network emulsion gels. This review follows the development of a double network by the possible combinations of proteins and polysaccharides (protein‐polysaccharide, double‐polysaccharide, and double‐protein), by analyzing their gelation mechanisms, interactions, and properties that are unique to their combination.

## 3 **Protein**‐**Polysaccharide Double‐Network Emulsion Gel Systems**

Various formulations of proteins and polysaccharides have been studied to create firm and stable emulsion gels. In most cases, polysaccharides were used as the gelling agent in a protein‐stabilized emulsion, in which protein does not form a continuous network within the polysaccharide gel. Conversely, protein‐only emulsion gels form a porous particulate gel (Dickinson 2012). The porous nature of protein gels rendered properties such as water holding capacity (WHC) and gel strength dependent on its microstructure, namely its aggregate size and uniformity (X. F. Wang et al. 2019). As the incorporation of polysaccharides into protein‐emulsion gel occupies the voids left by the protein network, the WHC, and mechanical strength may be improved. Table 1 shows current research on protein‐polysaccharide emulsion gels by identifying their structuring agent, gelation mechanism, and their interactions in the gel.

**TABLE 1:** Summary of protein‐polysaccharide network emulsion gel in research.

Structuring Biopolymer	Emulsifier	Gelation mechanism	Steps	Filler (A/I)	Properties	Reference
κC, PPI	PPI	Ca^2+^‐induced gelation of PPI and ion‐assisted coil‐helix transition of κC	1	A	↑G', texture, water holding capacity (0‐1.5% κC) Phase‐separation observed >0.75% κC Hydrophobic interactions and hydrogen bonds (PPI/κC)	(X. Li et al. 2024)
WPI‐EGCG, HAG	WPI‐EGCG Pickering particles	GDL/Ca^2+^‐induced gelation of WPI‐EGCG complex, Ca^2+^‐induced gelation of HAG	1	A	↑G’ (0.1–0.2% HAG) ↓G’ (0.3% HAG) Phase‐separation observed >0.2% HAG Hydrogen bond (HAG/HAG), ionic and hydrophobic interaction (HAG/WPI‐EGCG), disulfide bond (WPI‐EGCG/WPI‐EGCG)	(Qin et al. 2023)
PPI, κC	PPI	TGase‐induced crosslinking of PPI and Coil‐helix transition of κC	1	–	↑G’, texture (0–1.0%, κC>HAG >KGM) ↑Water holding capacity (HAG> κC = KGM) Phase separation observed at high polysaccharide content (HAG>>KGM> κC) Covalent bond (PPI/PPI), hydrogen bond (κC/ κC, HAG/HAG, KGM/KGM)	(Hou et al. 2022)
PPI, HAG		TGase‐induced crosslinking of PPI and Coil‐helix transition of HAG	1	–		
PPI, KGM		TGase‐induced crosslinking of PPI and swelling of KGM	1	–		
Zein, SA	Zein	TGase‐induced crosslinking of zein and Ca^2+^‐induced cross‐linking of SA	1	–	↑G’ (0–0.5% SA, +ve Ca^2+^, +ve TGase) ↑Emulsion stability, viscosity (+ve SA) TGase produced a weak zein gel but increased interfacial and emulsion stability Covalent bonds (zein/zein), ionic interactions (zein/SA, SA/SA)	(Yan et al. 2021)
WPC‐XG, κC	WPC‐XG complex	CA and K^+^‐induced aggregation of WPC‐XG complex and ion‐assisted coil‐helix transition of κC	1	A	↑G’, water holding capacity (0–0.8% κC, 0‐0.09% CA‐K^+^) ↑Freeze‐thaw stability (+ve κC, +ve CA‐K^+^) Electrostatic interactions (WPC‐XG/WPI‐XG), ionic interactions (κC/κC), hydrogen bonds (κC/WPC‐XG).	(Shen et al. 2024).
SPI, SA	SPI	Ca^2+^‐induced aggregation of SPI and Ca^2+^‐induced cross‐linking of SA	1	–	↑G’ (0–0.6% SA) ↓G’ (0.8–1.0% SA) ↑Water holding capacity, freeze‐thaw stability (0‐1.0% SA) Phase‐separation observed at high SA content (≥0.8% SA) Electrostatic interactions (SA/SPI), hydrophobic interactions (SPI/SPI), Ionic interactions, and hydrogen bonds (SA/SA)	(Wang et al. 2024)

Abbreviations: A: active, CA: citric acid, EGCG: (−)‐epigallocatechin‐3‐gallate conjugate, GDL: glucono‐δ‐lactone, HAG: high‐acyl gellan gem, I: inactive, κC: κ‐carrageenan, KGM: konjac glucomannan, PPI: pea protein Isolate, SA: sodium alginate, TGase: transglutaminase, WPC: whey protein concentrate, WPI: whey protein isolate.

### 3.1 Fabrication Process, Gelation Mechanisms, and Network Interaction

**FIGURE 2:**
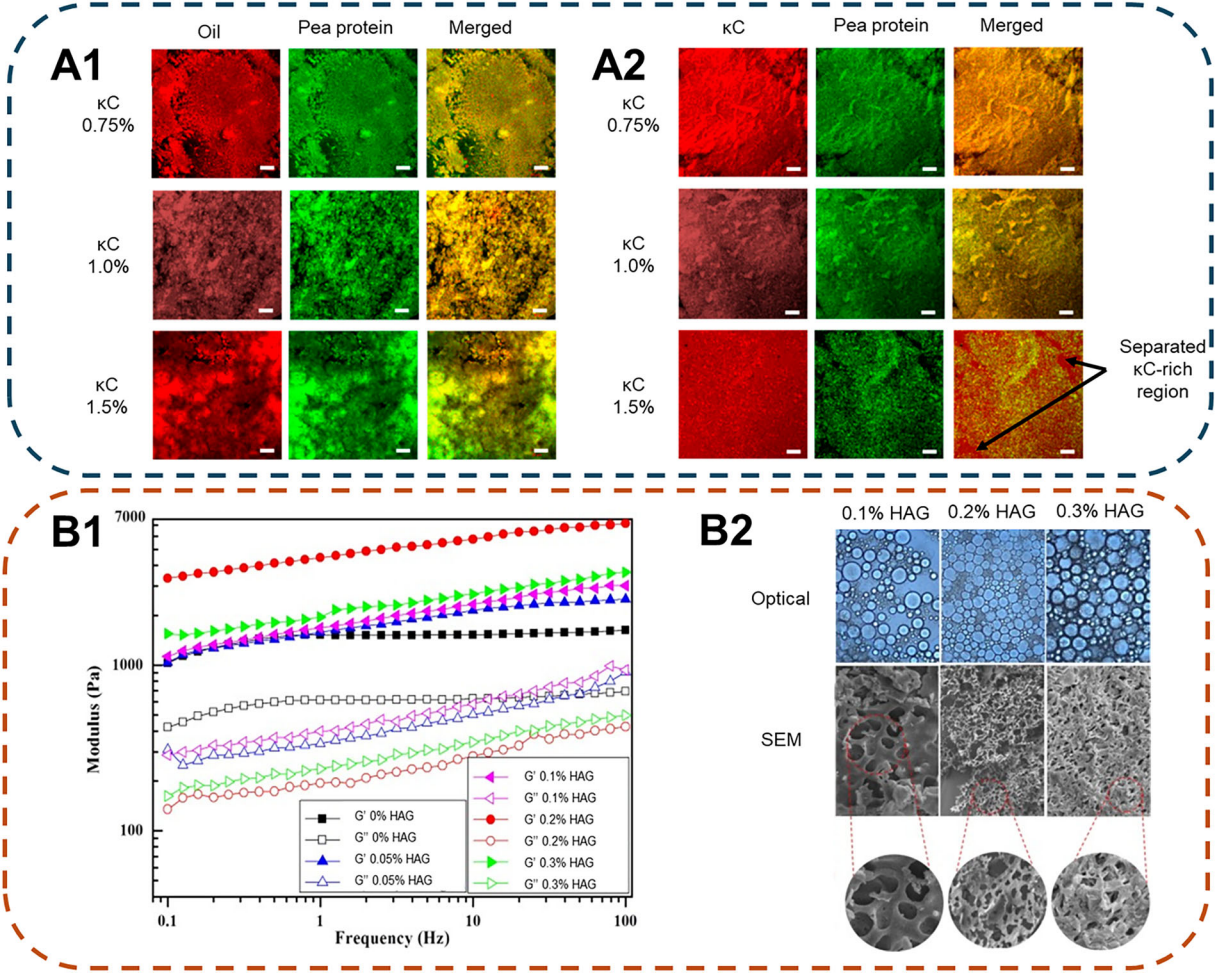
Microstructure and rheological properties of polysaccharide‐protein double network emulsion gel. (a) CLSM micrographs of a PPI‐κC double network gel from 0.75% to 1.5% κC (w/w). (a1) Micrograph showing the oil phase (red, Nile Red), pea protein (green, Nile Blue), and a merged image. (a2) Micrographs showing κC (red, fluorescein‐5‐isothiocyanate), pea protein (green, Nile Blue), and a merged image. *Source*: Reproduced from X. Li et al. (2024) under terms of CC‐BY license, Copyright 2024, MDPI. (b1) Frequency sweep of a WPI/EGCG‐HAG double network emulsion gel at varying HAG concentrations. (b2) Optical and SEM images of WPI/EGCG‐HAG double network emulsion gel with 0.1–0.3% (w/w) HAG. *Source*: Reproduced with permission from Qin et al. (Qin et al. 2023), Copyright 2022, Elsevier. Images were adapted.

Cold‐set double‐network gels using both protein and polysaccharide were routinely investigated as a carrier for bioactive ingredients. In a study by X. Li et al. (2024), pea protein isolate (PPI)/κC emulsion gels were created at varying κC concentrations (0.25–1.5%). Incorporating κC into the protein emulsion gel improved WHC and increased the gel's hardness, springiness, as well as chewiness of the gel. The intra‐ and internetwork interactions were determined by solvent dissociation. The gel was found to be guided by hydrophobic interactions (disassociated by EDTA) and hydrogen bonds (disassociated by SDS). However, the group determined that a double network could only be created with κC below 0.75%. As seen in Figure 2a, distinct phases of protein and κC locales were seen under confocal laser scanning microscopy (CLSM) and scanning electron microscopy (SEM) at high κC concentrations. This was attributed to the preferential binding of CaCl_2_ to κC, which, in high concentrations, curtailed the aggregation of pea protein, leading to the formation of a phase‐separated gel.

Qin et al. (2023) investigated the use of whey protein isolate (WPI) (−)‐epigallocatechin‐3‐gallate (EGCG) and HAG to form a Pickering double‐network emulsion gel for the encapsulation of probiotic bacteria *Lactobacillus plantarum*. Amphiphilic wettable particles of WPI and EGCG were formed by free‐radical induction to create Pickering emulsions that are resistant to enzymatic digestion. A one‐step method was used to create a double‐network emulsion gel by simultaneously adding GDL, CaCl_2,_ and HAG. As a result, two networks of GDL‐induced WPI/EGCG particulate gel and Ca^2+^‐induced HAG network were formed as the gel was allowed to set after 2 h. Overall, the existence of the double network improved the G’ of the emulsion gel, with maximum gel strength observed at 0.2% HAG (Figure 2b1). The simultaneous addition of GDL and CaCl_2_ was shown to encourage the aggregation of gelation of WPI/EGCG and HAG, respectively, with synergetic effects. Namely, this came in the form of acidification, which enhanced hydrogen bond formation in HAG alongside ion bridging of Ca^2+^, hydrophobic interactions between WPI/EGCG and HAG, and disulfide bonds between Pickering droplets. Nonetheless, excessive HAG (0.3%) was shown to have weakened the gel by showing a lower G’ compared to 0.2% HAG (Figure 2b1). It was suggested that this observation may be due to bridging flocculation, which could be the case as the gels’ microstructure becomes more compact as HAG content increases (Figure 2b2). This is not dissimilar to the findings from X. Li et al. (2024) (Figure 2a2), in which excessive polysaccharide (κC) caused phase separation. Thus, both studies indicate that achieving an optimal concentration of polymer is critical to obtaining maximum gel strength when salt/acid gelators are used.

A similar effect was seen in a covalently linked protein gel with a polysaccharide network, despite no competition in gelators. A double‐network emulsion gel system was created using TGase cross‐linked PPI and various polysaccharides (κC, HAG, and KGM) by Hou et al. (2022) using a one‐step method. PPI, along with a polysaccharide, was solubilized before forming an emulsion under high shear. The addition of TGase dispersed followed the emulsion formation, where the mixture was incubated at 37°C for protein covalent cross‐linking and later heated to 85°C for enzyme inhibition. Fourier transform infrared spectroscopy (FT‐IR) analysis of the emulsion gel revealed that interaction within the gel is guided by hydrogen bonds (broad absorption, 3100–3400 cm^−1^), and the addition of polysaccharides had a limited effect on the chemical cross‐linking of protein. Phase separation was again observed in samples prepared with high polysaccharide content at varying extents. The effect was the most pronounced with HAG, as large voids of presumed HAG‐rich areas were seen in its microstructure under CLSM across all concentrations tested (0.2–1% (w/w)) with larger oil droplets suggesting coalescence. Although a similar effect was seen in κC and KGM emulsion gels, the size of the voids was substantially smaller with well‐dispersed and uniform droplets. The effect was attributed to the thermodynamic incompatibility and volumetric effect between protein and polysaccharide at neutral pH. When a neutral polysaccharide such as KGM was added, changes in osmotic pressure from hydrophilic polysaccharides promoted the concentration and aggregation of the protein phase (Tobin et al. 2012). While at neutral pH or low ionic strength, repulsion occurs between the negatively charged protein and polysaccharide (Zhang et al. 2017).

The properties of proteins and polysaccharides allowed for the ready manipulation into forming distinctive networks within a single gel body. Double‐network in protein‐polysaccharide systems could take the benefit of both common (salt and acid) and distinctive gelators (enzymes) to form via a one‐step process during gelation. Interactions between networks by ionic and hydrophobic interactions were observed, especially in gel systems where common gelators were used. However, phase separation was observed for protein‐polysaccharide emulsion gels regardless of the type of gelator and bonds present in the gel. As phase‐separation was observed in gel created at higher polysaccharide concentrations and instances where preferential binding of ionic gelators to one species was observed, it implies that optimization of its formulation to ensure the greatest compatibility to create a synergistic network.

### 3.2 Properties of Protein‐Polysaccharide Emulsion Gel System

Polysaccharide‐protein double network emulsion gel possesses several advantages over single network gels. In a protein‐based emulsion gel, the formation of an additional polysaccharide network improves gel strength and WHC beyond that achieved by the single network (Table 1). Generally, an increase in polysaccharide concentration would increase the WHC of a gel as the pores within the particulate gel network are filled by hydrophilic polysaccharides. WHC in double‐network emulsion gel may reach >90% at higher polysaccharide concentrations (Hou et al. 2022; X. Li et al. 2024; Zhang et al. 2024). Much like single‐network systems, WHC of double‐network gels may also depend on oil volume and the type and amount of gelator used (Shen et al. 2024; Zhang et al. 2021). As the principal component for inducing cross‐linkage between molecules, an increase in the gelator concentration may enhance cross‐linkage, resulting in a denser gel (Shen et al. 2024). An increase in oil content increases water retention within the gel as the compacting oil droplets enhance capillary effects and resistance to mechanical forces from the droplet's elasticity (Line et al. 2005). A similar effect was seen in wheat bran arabinoxylan and soy protein isolate (SPI) emulsion gel up to 10% (w/w) oil. Beyond that point, the increase in oil content led to a decrease in WHC as phase separation was amplified (Zhang et al. 2021).

The formation of a protein‐polysaccharide double network emulsion gel has also seen improvements in gel strength and texture. Increases in biopolymer content were generally seen to positively affect gel strength and the resulting texture of the gel as measured in texture profile analysis (Hou et al. 2022; Zhang et al. 2024). For instance, the inclusion of κC (0.25–1.5% κC) in PPI emulsion gel yielded 1.09 to 74.45 times hardness improvement compared to 0% κC. This indicates that the addition of polysaccharides is a viable pathway to further texturize protein gels, thus improving the perceived texture of these systems. A maximum G’ in small amplitude oscillatory strain analysis was observed in samples containing an intermediate gum content in systems with gellan, inulin, and SA (Qin et al. 2023; Wang et al. 2024; Q. Q. Xu et al. 2021). The effect was attributed to the disruption of gel microstructure as phase separation was enhanced at high polysaccharide concentration. The preparation method and the role of oil droplets within the double network were also seen to affect the final mechanical properties. Using Tween 80 as the emulsifier in a casein and SA system decreased the extent of phase separation (Li et al. 2021). As phase separation happens in samples without Tween 80, a maximum G’ and firmness were observed at an intermediate alginate content of 0.4% (Li et al. 2021). The use of an SME like Tween 80 enabled an emulsion to be created in the presence of alginate, which thickens the emulsion and impedes the coalescence of oil droplets (Li et al. 2021). Even as oil droplets predominantly exist within the casein phase, the Tween 80 stabilized emulsion was still observed to minimize phase separation and, hence, allow the hardness of the gel to increase (Li et al. 2021).

The creation of a polysaccharide‐protein double network gel was often able to achieve properties beyond those of a single network. The difference in interactions between protein and polysaccharide was easily exploited to readily form a double network. Nevertheless, it was also the difference in interactions that led to phase separation. The compatibility of biopolymers appeared to be the determining factor governing the properties of the protein‐polysaccharide double network. The compatibility in a mixture is determined by pH, ionic strength, net charge of each biopolymer, and hydrophobicity demonstrated by various studies (Tobin et al. 2012; Wang et al. 2024; Zhang et al. 2017). The model on binary protein and polysaccharide solutions by Grinberg and Tolstoguzov (1997) appeared to apply to double‐network emulsion gel. Where only a slight net repulsive protein and polysaccharide are required for a bi‐phased microstructure to appear at low total biopolymer concentration (Dickinson 2003; Grinberg and Tolstoguzov 1997). Therefore, while polysaccharides acted as a filler to form a dense combined network that could ensure the stability of the emulsion gel, the overall mechanical strength of the emulsion gel is highly dependent on the compatibility of the two biopolymers. The management of the interaction via the selection of biopolymer and the concentration of gelator(s) is critical in modulating the characteristics of the gel. All in all, the polysaccharide‐protein double‐network structured emulsion remains a robust schematic for emulsion gel design.

## 4 Double Polysaccharide Emulsion Gel Systems

Emulsion gels structured by a double polysaccharide network have garnered interest among researchers aiming to create firm and resilient emulsion gels. The creation of a second network offers flexibility in manipulating the thermal properties of the emulsion gel. Polysaccharides were adopted into various configurations within the emulsion gel. Unlike the previously described protein‐polysaccharide system, where one of the structuring agents could be readily used as an emulsifying agent, specific polysaccharides with surface‐active properties and Pickering particles were used for emulsion stabilization. This section explores the current state of research on double polysaccharide emulsion gels, focusing on processing, gelation mechanisms, and properties enhanced through combining polysaccharides. Table 2 shows current research on double‐polysaccharide emulsion gels by identifying their structuring agent, gelation mechanism, and their interactions in the gel.

**TABLE 2:** Summary of double polysaccharide network emulsion gels in research.

Structuring Biopolymer	Emulsifier	Gelation mechanism	Steps	Filler (A/I)	Properties	Reference
KGM, XG	Tween 80/KGM‐XG	Synergistic gelation of KGM and XG	1	I/A	↓ Gel strength (0–40% oil (w/w), KGM‐XG) ↓↓ Gel strength (0–40% oil, KGM‐tween‐XG) Type A interaction, XG helix attached to KGM chain. Tween‐80 concentration was above the critical micelle concentration, creating an inactive filler.	(Yang et al. 2019)
Methylcellulose, DKG	Methylcellulose	Heat‐induced gelation of MC and DKG	1	–	G’_max_ (20°C) at 6:4 DKG:MC, G’_max_ (80°C) at 2:8 DKG:MC Mixing ratio does not affect WHC DKG limited thermal reversibility of MC Hydrogen bond and hydrophobic interactions (DKG/MC at oil interface), hydrogen bond (DKG/MC far from interface)	(Jeong et al. 2023)
SA, agar, SPI	SPI	Coil‐helix transition agar, Ca^2+^‐induced gelation of SA.	2	A	↑G*, water holding capacity, freeze‐thaw stability (0–1% (w/w) SA) G* remained steady during heating (10–90°C, SA ≥0.25%) Ionic interactions (SA/SA), hydrogen bond (SA/agar).	(Choi et al. 2023)
SNC, SA	SNC, SA	SNC‐SA‐co‐stabilized droplet aggregation, swelling of SA.	1	–	↑ G’ (0–0.5% SA), ↓ G’ (1.0% SA) ↑ emulsion stability (0‐0.5% SA, 20–60% oil) Hydrogen bond (SNC/SA)	(Cai et al. 2024)
SA‐LAG, CMCS, OSA starch	OSA starch	Ca^2+^‐induced gelation of SA and LAG	1	–	↑ Hardness, Young's modulus (0.2–0.8% LAG) Ionic interactions (SA/SA, SA/GG, CMCS/CMCS), hydrogen bond (SA/GG/CMCS)	(Zheng et al. 2022)
WPC, κC, SA	WPC	Coil‐helix transition of κC and swelling of SA.	1	–	↑ Hardness, Water holding capacity Oil droplet flocculation at high SA content (1.0 g/100 mL SA) Hydrogen bond (SA/κC), electrostatic interactions (SA/WPC).	(Liang et al. 2024)
SA, KGM. Egg yolk	Egg yolk	Swelling of KGM and SA	1	A	↑G’, freeze‐thaw stability (0.5–4.0% (w/v) KGM, 5–30% oil) KGM enhanced thixotropic properties of the SA gel	(Yang et al. 2020)

Abbreviations: A: active, CMCS: carboxymethyl chitosan, DKG: deacetylated konjac glucomannan, I: inactive, κC: κ‐carrageenan, KGM: konjac glucomannan, LAG: low‐acyl gellan gum, OSA‐starch: octenyl succinic anhydride modified starch, SA: sodium alginate, SNC: starch nanocrystal, SPI: soy protein isolate, WPC: whey protein concentrate, XG: xanthan gum.

### 4.1 Fabrication Process, Gelation Mechanisms, and Network Interaction

Synergistic gelation of polysaccharides has been extensively explored by researchers between gelling and nongelling polysaccharides. A combination of polysaccharides was shown to induce gelation at a lower polysaccharide concentration that would otherwise not gel (Nishinari 2021). Extending the understanding to emulsion gels, Yang et al. (2019) explored the use of xanthan and KGM with and without an SME (Tween‐80). The xanthan‐KGM gel was created through a one‐pot, one‐step method where xanthan/KGM/KGM‐Tween‐80 was used to emulsify the oil phase before mixing with KGM/xanthan (depending on the initial emulsifier) in a heating and cooling cycle. A Type A interaction was found to be the dominant interaction between KGM and xanthan in the emulsion gel (Yang et al. 2019). Type A interaction is characterized by a low gel–sol transition temperature between 30–45°C due to the retention of the xanthan helical structure when interacting with KGM (Abbaszadeh et al. 2016). The emulsifying capacity of the individual polysaccharide and the inclusion of SME were critical to the stability and stiffness of the final gel in this regime. Due to the lower emulsifying capacity of xanthan, the xanthan‐KGM format displayed a weaker gel structure than its KGM‐xanthan counterpart, as the former was prone to destabilization. The inclusion of an SME was effective in creating uniform droplets that were smaller in size. However, as the concentration of Tween‐80 was above its critical micelle concentration, the dispersed phase behaved as an inactive filler. This manifested in the weakening of the gel in stiffness and strength as oil content increased.

A thermal‐irreversible emulsion gel using methylcellulose (MC) and DKG was created by Jeong et al. (2023). In a binary mixture of DKG and MC, oil was added and homogenized to form an emulsion before the gel was heat‐set at 80°C for 30 min in a one‐pot, one‐step method. In this configuration, MC and, to a certain extent, partially deacetylated KGM were responsible for both emulsion stabilization and gel structuring due to their amphiphilic nature and gelling properties by hydrophobic interactions upon heating. Unlike DKG, which forms a thermal‐irreversible gel upon heating, MC gels are thermal‐reversible and soften upon cooling (Kobayashi et al. 1999). Individually, FT‐IR analysis shows that hydrogen bonds were responsible for DKG cross‐linkage, and MC was predominantly linked by hydrophobic interactions within the gel matrix. Both hydrogen and hydrophobic interactions were found at the interface as droplets were stabilized by DKG and MC. In contrast, hydrogen bonds were the dominant force guiding inter‐network interactions between MC and DKG far from the interface.

**FIGURE 3:**
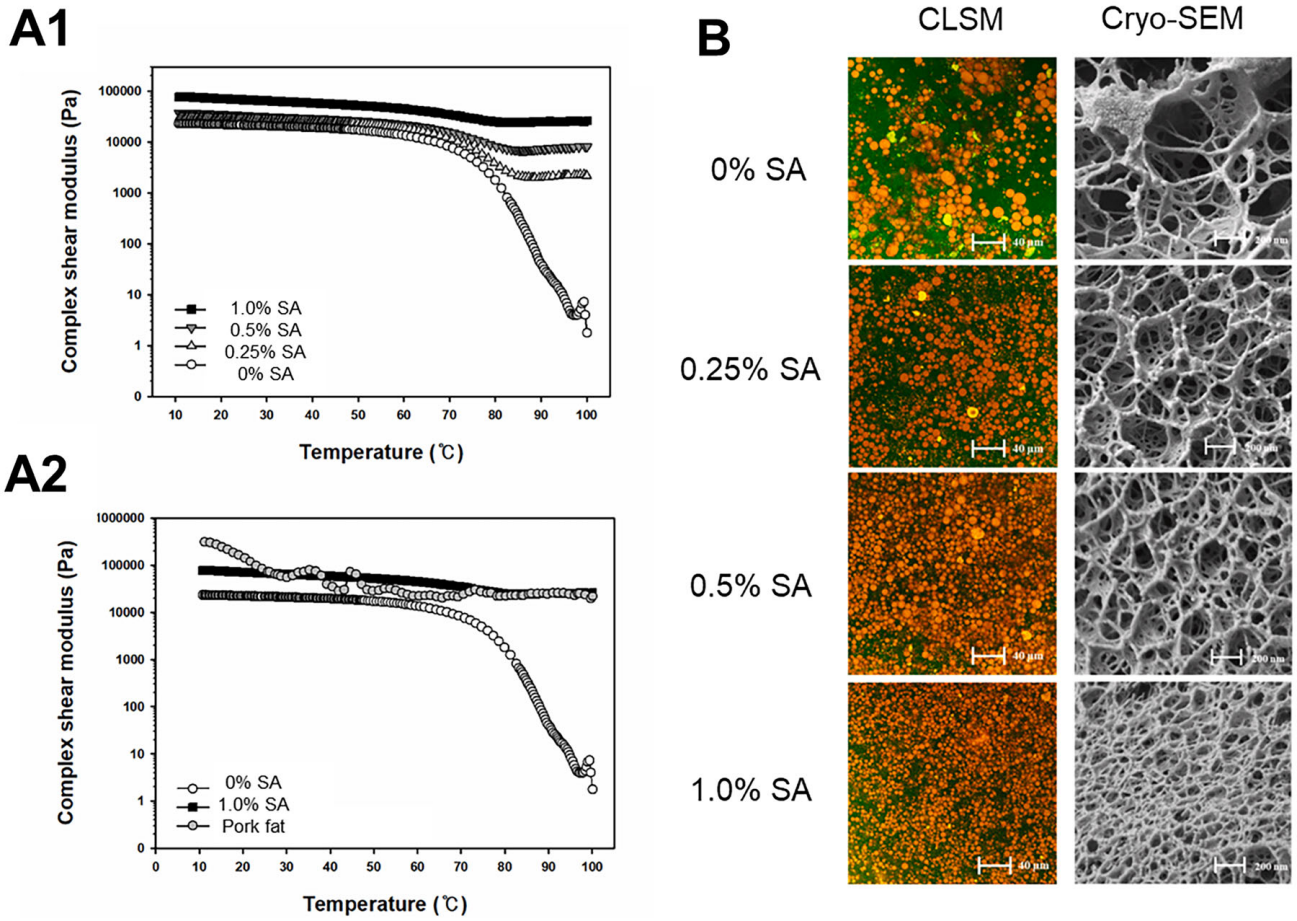
Rheological properties and the microstructure of an agar‐SA emulsion gel reported by Choi et al. (2023). (a) Complex shear modulus (G*) of emulsion gels at varying temperatures (a1) with varying SA content. (a2) Comparison with pork fat. (b) CLSM and cryo‐SEM micrographs at varying SA content. *Source*: Reproduced with permission from Choi et al. (2023), Copyright 2023, Elsevier.

Combining surface active polysaccharides with protein may enhance the gel properties of a double polysaccharide emulsion gel system. Choi et al. (2023) reported a double‐network emulsion gel comprising SPI, alginate, and agar to create a thermal‐irreversible emulsion gel (Figure 3a). The gel was created through a two‐step method where an initial agar‐structured emulsion gel was first created before immersion into a CaCl_2_ solution for alginate cross‐linking. As a result, two networks of assembled agar helix and Ca^2+^ mediated alginate network were formed. Hydrogen bonds formed the guiding interactions between the two matrices due to the abundance of hydroxyl groups on both agar and alginate. Notably, an increase in polysaccharide content did not cause phase separation (Figure 3b) or detriment to the mechanical properties of the gel (Figure 3a). Alginate was used as one of the structural components with a stabilizing effect on the dispersed oil droplets due to its ability to adsorb at the oil/water interface of food oils itself and especially through electrostatic interactions with protein molecules adsorbed at the oil/water interface (Lü et al. 2020; Su et al. 2018). As alginate was partially responsible for the stabilization of oil droplets, the droplets could behave as an active filler within the gel matrix. Thus, an increase in gel mechanical properties was observed for alginate content up to 1% (w/w) (Figure 3a). Unlike the SME used in the study by Yang et al. (2019), both SPI and alginate were able to participate in the emulsification process in creating a rigid gel even as polysaccharide content increased.

The formation of Pickering particles has proven to be a robust method to negate the use of surfactants in polysaccharide‐based systems (Cai et al. 2024; Jiang et al. 2019; Li et al. 2023; Zheng et al. 2022) Starch is one of the most utilized polysaccharides due to its cost‐effectiveness and GRAS nature (Zhu 2019). In order to stabilize emulsion, most starches require modification due to their hydrophilic nature. Modifications such as octenyl succinic anhydride modification (OSA), hydrolysis, and milling are common methods to produce starch‐based Pickering particles. Cai et al. (2024) explored the use of alginate to improve the stability and mechanical properties of starch nanocrystal Pickering emulsion gel. Alginate and starch nanocrystals partook in the co‐stabilization of the oil interface with the two polysaccharides interacting through hydrogen bonds. The viscosity‐inducing effect of alginate was found to be critical to the rheological behavior of the gel, inducing a solid‐like rheology and increased stability. Apart from co‐stabilization, the use of starch as a Pickering particle embedded within a polysaccharide matrix is also attractive. Starch particles were able to interact with an external polysaccharide matrix, as shown by Zheng et al. (2022). Despite the chemical modification, OSA‐starch could interact through hydrogen bonds and electrostatic interactions with the alginate–gellan–carboxymethyl chitosan matrix while stabilizing the dispersed oil phase. Particularly, the electrostatic interaction and matrix‐filling effect of the droplets provide resistance to swelling.

Among the regimes studied for double‐polysaccharide emulsion gel, it was apparent that the formation of synergistic polysaccharide networks could improve the stability, mechanical properties, and thermal behavior of single‐network emulsion gels. Mechanically, polysaccharides were shown to readily form intra‐ and internetwork hydrogen bonds, which greatly improved mechanical properties. Moreover, the mixing of a thermal reversible and a thermal irreversible polysaccharide was able to create the desired rheological profile over a range of temperatures. The relationship between the dispersed oil phase and the gel matrix was observed to be determined by the emulsifying capacity of individual polysaccharides or the inclusion of an emulsifier. Depending on the choice of emulsifier and structuring polysaccharide, the dispersed phase may be either inactive or active.

### 4.2 Properties of Polysaccharide‐Based Emulsion Gel Systems

The dense gel created by polysaccharides has demonstrated excellent WHC and freeze‐thaw stability (Table 2). The WHC double‐polysaccharide emulsion gels were observed to be typically above 90% (Choi et al. 2023; Jeong et al. 2023; Liang et al. 2024; Yang et al. 2020). A progressive increase in the concentration of the second polysaccharide improved the freeze‐thaw stability of the emulsion gel. An addition of 1% alginate into an agar/alginate emulsion gel matrix was observed to reduce syneresis by >50% (Choi et al. 2023). Droplet coalescence was not observed in alginate/KGM‐based emulsion gels for oil content up to 30% (w/w) (Yang et al. 2020).

The mechanical properties of double‐polysaccharide networks were shown to be superior to those of a single network (Choi et al. 2023; Yang et al. 2020). Gel strength as measured in rheological and texture analysis (compression/penetration) of the emulsion gel was increased as a second polysaccharide was introduced (Choi et al. 2023; Yang et al. 2019; Yang et al. 2020). A 2.57× increase in gel hardness and 1.27× increase in chewiness was recorded in the alginate/agar emulsion gel devised by Choi et al. (2023). Between xanthan and KGM, a maximum strength was obtained at a ratio of 4:6, with the strength being greater than the sum of samples prepared with only either polysaccharide. The creation of two individual networks is a strategy to mitigate temperature‐dependent properties. A temperature sweep of an alginate/agar gel revealed that the gel remained irreversible at increasing temperatures with the introduction of alginate (Choi et al. 2023). In samples prepared with no alginate, the complex shear modulus (G^*^) was reduced by at least three magnitudes when the gel was heated to 100°C, while samples with alginate remained steady (Figure 3a). Thermal dependency at lower temperatures, like that of MC, could be further mitigated through the use of fat instead of oil (Jeong et al. 2023). This enables these double‐polysaccharide systems to be used in processed food, as cooking would not deform their structure.

Phase separation was observed to a lesser degree in double‐polysaccharide emulsion gels than in protein‐polysaccharide systems. Several factors may have contributed to this issue. Polysaccharide‐based emulsion gels using two hydrophilic polysaccharides may use SME for emulsion stabilization (Yang et al. 2019). In these cases, the network does not interact with the droplet, hence limiting incompatibility. A similar case with protein‐polysaccharide double‐network systems was also observed (Li et al. 2021). An increase in viscosity during the dispersion of polysaccharides was also believed to contribute to the stability of droplets within the gel matrix. An increase in viscosity reduces coalescence and droplet size as shear forces are increased during mixing (Choi et al. 2023). The use of surface‐active polysaccharides and a protein‐based emulsifier blend may allow oil droplets to behave as an active filler, thus limiting phase separation. Nonetheless, only a small amount of polysaccharide would be involved in droplet stabilization. Studies have suggested that this effect may be at the helm of the interaction between the biopolymers and their respective concentration. Although agar/alginate and SPI (1% and 3%) were not found to undergo phase separation, alginate, and casein (0.75% and 8%) were readily separated (Choi et al. 2023; Li et al. 2021). It was suggested that the difference in amphiphilicity, where alginate is substantially more hydrophilic than casein, was responsible for their differences (Li et al. 2021; McClements and Jafari 2018).

Although not unique to polysaccharide‐only systems, the use of polysaccharides as an emulsifier and structuring agent has proven to be effective in controlling and directing the release of oil or other encapsulated ingredients. For instance, the use of starch also gives rise to the potential of directing the release of oil during mastication. A modified starch/gellan gum emulsion gel was created by Hu et al. (2021) to study the difference in oil release between WPI‐ and starch‐emulsified droplets. As α‐amylase was able to digest the emulsifier, using modified starch was effective in increasing oil release during oral processing. The increase in oil release was accompanied by an increase in oily perception. At 5% oil (w/w), modified starch/gellan emulsion gel was able to achieve a similar oiliness to 20% WPI/gellan emulsion gel. The findings may be used in creating low‐fat food with little compromise on sensory attributes. On the other hand, the release of nutrients may also be modulated through the use of different polysaccharides. In β‐carotene‐loaded Pickering emulsions, the high viscosity of KGM was effective in creating a sustained release regime as it impedes the contact between the oil droplets and digestive fluids (Xu et al. 2023).

The success of a double‐polysaccharide emulsion gel design depends on multiple variables that are unique to this regime. Overall, double‐polysaccharide emulsion gels are an effective way to create highly texturized foods. These polysaccharide‐based networks demonstrated high stability and strength against stressors, mainly due to their hydrophilic nature, which forms a compact gel structure. Again, due to the mostly hydrophilic nature of polysaccharides, the choice of emulsifier (whether SME, protein, or polysaccharide) will influence the stability, mechanical properties, as well as the nutritional profile of the emulsion gel. Thus, this makes the selection of emulsifier in a double‐polysaccharide system a critical step to be modulation optimized for the specific use of the emulsion gel.

## 5 Double Protein Emulsion Gel Systems

Protein‐based emulsion gel was among the first types of emulsion gels created, as protein could be both a structuring agent and an emulsifier. The desire to use protein in novel food development stems from the nutritional importance of protein in our diet and its associated positive consumer perception (Aschemann‐Witzel and Peschel 2019). However, difficulties exist in creating double‐network mixed protein gels. The mechanical properties of a binary protein gel are determined by the interactions between the protein molecules and their respective interaction with water (Nicolai 2019). Furthermore, similarities between proteins and the mostly nonspecific nature in inducing gelation created mixed results. This stands in contrast to the previously discussed protein‐polysaccharide and double‐polysaccharide systems. Nonetheless, the prospect of a protein‐based product remains enticing from the better consumer acceptance alone. To meet this end, recent studies explored blends between various sources (plant and animal) and classes. Table 3 shows current research on double‐protein emulsion gels by identifying their structuring agent, gelation mechanism, and their interactions in the gel.

**TABLE 3:** Summary of double‐protein network emulsion gels in research.

Structuring Biopolymer	Emulsifier	Gelation mechanism	Steps	Filler (A/I)	Properties	Reference
WPI	WPI	TGase‐induced crosslinking and Ca^2+^‐induced gelation of WPI	1	A	Double cross‐linked WPI emulsion gel ↑G’, hardness, WHC, F‐T stability (+ve oil, +ve Ca^2+^, +ve Ca^2+^‐TGase) TGase has a limited effect on G’ and hardness but increased WHC and F‐T stability Covalent bond, ionic interactions (WPI/WPI)	(Liang et al. 2020)
SPI, WPI	SPI/WPI	Heat‐induced gelation of WPI and SPI Ca^2+^‐induced and Heat‐induced gelation of WPI and SPI	1	A	G’ and hardness of mixed samples lie between pure WPI and SPI emulsion gel, depending on their ratio ↑WHC (0.1–0.5 mol/L Ca^2+^) No distinctive networks, SPI may be active fillers	(Cheng et al. 2024; X. Zhang et al. 2022)
MCN, PPI/SPI	MCN‐PPI/ MCN‐SPI	Heat‐induced gelation of MCN and PPI/SPI	1	A	↑G’ (0‐15% Oil, 1–4% total protein) Ca_3_(PO_4_)_2_ from MCN led to some Ca^2+^ ionic bridges between proteins Lower protein content required for gelation as oil causes protein structure changes. Distinctive MCN and PPI/SPI network	(Schmitt et al. 2019; Silva et al. 2019)
SPI, EP	SPI‐EP	TGase‐induced crosslinking of SPI and heat‐induced gelation of EP	2	A	↑G’, hardness, chewiness, and WHC (0–20% oil) ↓ Springiness and cohesiveness (0–20% oil) Oil type had little influence on gel properties	(Zhang et al. 2020b)
WPF, FG	WPF‐FG/WPI‐FG	Coil‐helix transition of FG	1	–	WPF decreases gelation time of FG at low pH (pH = 3) WPF‐FG has increased deformation resistance compared to WPI‐FG Hydrophobic interactions (WPF/FG)	(Lin et al. 2023)
PNP, HGRF	PNP	GDL‐induced gelation of PNP	1	–	Highest G’, F‐T stability at 2:1 PNP: HGRF Nanofibrils fill the void between oil droplets and adsorb at the O/W interface Electrostatic interactions (PNP/PNP), hydrophobic interactions (PNP/HGRF)	(Kong et al. 2024)

Abbreviations: A: active, EP: egg protein, FG: fish gelatin, F‐T: freeze‐thaw, HGRF: hydrolyzed rice glutelin fibril, I: inactive, MCN: micellar casein, PNP: pea protein nanoparticles. PPI: pea protein isolate, SPI: soy protein isolate, TGase: transglutaminase, WHC: water holding capacity, WPF: whey protein fibrils, WPI: whey protein isolate.

### 5.1 Fabrication Process, Gelation Mechanisms, and Network Interaction

A study to understand the physical characteristics of a double‐crosslinked protein emulsion gel was conducted by Liang et al. (2020). A WPI‐stabilized emulsion was crosslinked by Ca^2+^ and TGase in a one‐pot one‐step method involving the simultaneous addition of the two gellators and subsequent incubation at 37°C for 4 h. A dense and uniform gel was observed under CLSM and SEM due to the double‐cross‐linkage. Between TGase and Ca^2+^, the latter was seen to have increased protein aggregation as the ion is not sub‐unit specific. FT‐IR analysis revealed the formation of both strong amide covalent bonds (1651 and 1746 cm^−1^) and salt bridges between protein molecules (1651 cm^−1^), which contributed to the structure of the emulsion gel. The protein structure was also rearranged by double‐cross‐linkage. The simultaneous addition of the two gelators was seen to increase α‐helix and decrease β‐turns within the emulsion gel compared to those formed by either. All of these collimated to enhanced mechanical properties (G’ and hardness). The oil phase was identified as an active filler from the dual role protein plays as the emulsifier and structural agent. Thus, the mechanical properties were improved compared with double‐crosslinked WPI hydrogels. Although the double‐cross‐linked network formed by a single protein, albeit through two means, may not seem as distinctive as a double network formed by protein/polysaccharide or polysaccharide/polysaccharide, the study underscored the potential of manipulating proteins to develop protein‐only structures.

Amongst common proteins used to construct emulsion gels, a composite SPI and WPI emulsion gel was created by X. Zhang et al. (2022) and Cheng et al. (2024). The composite gel was created through a one‐pot, one‐step method where the final gel was induced by heat. CaCl_2_ was also included in the formulation by X. Zhang et al. (2022) before heating. The inclusion of a dispersed oil phase did not impact the interactions between WPI and SPI, as it was shown by Jose et al. (2016). Within a heat‐induced WPI/SPI composite emulsion gel, WPI and SPI were not able to form distinct networks, with SPI largely impeding the formation of a dense WPI network when used at a high ratio (Cheng et al. 2024). Like its hydrogel counterpart, both the rheology and textural quality of the composite emulsion gel were found to be between pure WPI and SPI emulsion gel at a constant protein content. However, the G’ of SPI/WPI composite emulsion gel was greater than the sum of the G’ recorded by the individual gel at their respective ratio (2.15x at 1:1 WPI/SPI). Since SPI requires a higher protein content to form a self‐supporting gel, along with SPI and WPI differ in denaturation and gelation temperature. SPI was believed to have acted as an active filler in the whey protein network, particularly at a lower SPI ratio. Evidently, both similarities and differences between SPI and WPI, particularly gelation mechanisms and conditions, underpin the difficulty in creating a true double network between the two species.

**FIGURE 4:**
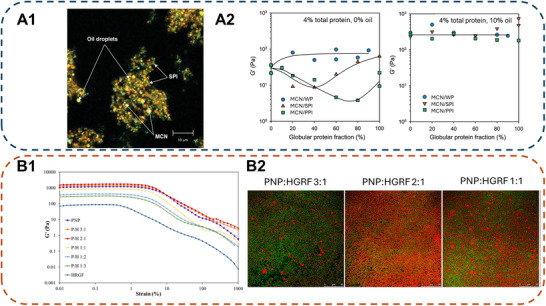
Rheological properties and microstructure of double‐protein emulsion gels. (a1) CLSM of SPI/MCN emulsion gel. The oil droplets were stained by Nile Red, with SPI and MCN being specifically labeled with antibodies. (a2) G’ of hydrogel (0% oil) or emulsion gel (10% oil) at varying globular plant protein fractions with 4% total protein content (MCN with PPI, SPI, or WP). *Source*: Reproduced with permission from Schmitt et al. (2019) and Silva et al. (2019), Copyright 2019, Elsevier and Copyright 2019, Elsevier). (b1) Amplitude sweeps of PNP/HGRF emulsion gel at different PNP: HGRF ratios. (b2) CLSM micrographs of PNP/HGRF emulsion gels at varying ratios (oil: red, protein: green). *Source*: Reproduced with permission from Kong et al. (2024), Copyright 2024, Elsevier.

Various emulsion gels by blending different classes of proteins such as plant prolamin‐globulin, plant globulin‐casein, and ovalbumin‐plant globulin were investigated by researchers. Silva et al. (2019) reported that the replacement of micellar casein (MCN) by plant protein (soy or pea) in a heat‐set emulsion gel yielded comparable gel firmness to MCN‐only samples. CLSM observations with individual protein labeling revealed that soy protein and MCN formed distinctive networks rather than co‐gelation (Schmitt et al. 2019) (Figure 4a1). Oil was seen to be embedded within the protein matrix and acted as an active filler contributing positively to its stiffness. At 4% total protein content (MCN with PPI, SPI, or whey protein (WP)) and 10% oil content, the emulsion gel created negated the antagonistic effect between the plant proteins and MCN within an aqueous dispersion (Figure 4a2). This indicated that the protein interaction at the O/W interface is more significant than those further removed from the interface (Silva et al. 2019).

Glusac et al. (2018) reported the creation of a tyrosinase cross‐linked zein and potato protein emulsion gel. Tyrosinase crosslinked emulsion gel was seen to have a greater G’ than noncrosslinked emulsion gel by 2.5 orders of magnitude with substantial improvement in stability. SDS‐PAGE patterns revealed that tyrosinase was responsible for the polymerization of potato protease inhibitors and α‐zein fractions. It was assumed that both proteins participated in the covalent gel network, indicating that synergy exists between potato and zein proteins. Despite the improvement achieved, limited evidence showed that two independent networks existed in the current gel. Moreover, the role of potato patatin is largely unknown in the current study. Potato patatin may be utilized to form a secondary network, owing to its gelation properties and emulsifying potential (Schmidt et al. 2019). As patatin makes up ∼40% of the total protein content, further involvement may be beneficial in unlocking the full potential of the protein blend (Schmidt et al. 2019).

A study by Zhang et al. (2020a) investigated the effect of oil type and content on a combined egg protein with SPI in a TGase cross‐linked emulsion gel. A one‐pot, two‐step method was reported where sequential gelation was conducted by incubation at 40°C and then at 85°C for the complete gelation of the two proteins. As established by their previous study, it was understood that two networks may have been created within the gel matrix. In the gel, TGase was found to primarily crosslink SPI with a heat‐induced egg protein gel interspersed within (Zhang et al. 2020b). An active dispersed oil phase was seen as protein participated as both the emulsifier and structure agent. This was marked by an increase in G’ and hardness of the gel as oil content increased from 5–20% (w/w). A denser and more uniform gel network was observed as oil content increased. The abundance of oil droplets encouraged the unfolding and rearrangement of protein, in turn, promoting enzymatic cross‐linkage. The type of oil (olive, soy, and menhaden) did not have a significant impact on the gelation mechanism and properties of the protein‐based emulsion gel.

Recent advancements in utilizing protein fibrils may prove useful in creating double protein network gels. The fibrilization of globulin protein typically takes place at pH substantially below the isoelectric point and at high temperatures, where protein self‐assembles into amyloid‐like strands by hydrophobic interactions. These fibrils were reported to minimize allergenicity and improve emulsion stability by reducing aggregation and flocculation through droplet entanglement (Xu et al. 2024). An emulsion gel using whey protein fibrils (WPF) and fish gelatin (FG) was created by Lin et al. (2023). The group reported that WPF‐FG emulsion gel possesses several advantages over WPI‐FG and FG‐only gels, especially at low pH. WPF was seen to accelerate the gelatin gelation by promoting coil‐helix transformation and thus provide rapid stabilization at pH 3. Kong et al. (2024) proposed a Pickering emulsion gel using pea protein nanoparticles (PNP) and hydrolyzed rice fibrils (HRGF) through a one‐step gelation process. The group reported that a 2:1 (PNP: HRGF) ratio was optimal to achieve superior mechanical properties. At this ratio, the G’ under large amplitude oscillatory shear analysis of the emulsion gel is the greatest and greater than that of PNP‐only and HRGF‐only gel (Figure 4b1). This was supported by the Lissajous plot, where, despite showing rectangular distortion, the stress of the 2:1 ratio sample is the greatest among all other ratios tested. A smaller droplet size as well as an increase in hydrophobic interactions (FT‐IR, peak shift from 2924 to 2923 cm^−1^) between the PNP and HGRF networks was attributed to the observed result (Figure 4b2). Furthermore, HGRF may have also acted as a filler between voids, along with the hydrophobic interactions, added to the mechanical properties of the gel (Kong et al. 2024).

The use of protein blends yielded mixed results in creating an emulsion gel with two distinctive protein networks. The interactions between proteins and their interactions with the gelator during gelation were instrumental in determining the final gel matrix. Successful double‐network formation was observed with proteins that showed preferentiality with their gelator. This was the case for the TGase‐induced soy and egg protein mix. Discrepancies in gelation condition and required concentration contributed to the malformation of two distinctive networks in heat‐set WPI/SPI emulsion gels. Prefabricated protein fibrils may offer another means of creating double networks, as it appears that protein fibrils have limited interference in the formation of the second protein network. Nonetheless, the dual role of protein remains beneficial in emulsion gel design. Differing from polysaccharide‐protein emulsion gel, weakening due to phase separation was not observed in double‐protein gels. The active role of oil means that characteristics allow for tunable gel characteristics as well as the suitability of creating stable high‐oil content products.

### 5.2 Properties of Protein‐Based Emulsion Gel Systems

Like the systems examined previously, a double protein system improves the gel strength and stability of a single network emulsion gel. A double‐network, as well as double‐crosslinked emulsion gel, possesses a denser gel network that has increased water retention and resistance to deformation (Table 3). As an active filler, the inclusion of oil reinforces these properties (Silva et al. 2019; Zhang et al. 2020a). For instance, an oil content increase from 0% to 20% (w/w) translated to an increase in hardness for TGase‐induced SPI‐egg emulsion gel. In comparison with other double‐network systems, protein‐only systems have the benefit of carrying a high oil content without obvious destabilization (Zhang et al. 2020a). Nonetheless, current double‐protein gels typically showed weaker gel strength at G’ ≈1×10^3^ Pa compared with other double‐network systems, where G’ could reach up to 10^6^ Pa (Choi et al. 2023; Hou et al. 2022; Kong et al. 2024; Liang et al. 2024; Qin et al. 2023; Silva et al. 2019). On the other hand, the dense gel that was created by two distinctive networks or double‐cross‐linkage was able to demonstrate a high WHC (>80%) (Li et al. 2022; Liang et al. 2020; Zhang et al. 2020a). Similarly, freeze‐thaw stability was also improved when protein was double‐crosslinked with TGase and Ca^2+^. In double‐crosslinked gels, the syneresis was the lowest, with the rate gradually decreasing over cycles. Despite the desirable stability, a weaker gel remains a limiting factor for the adoption of these double‐protein emulsion gels.

In comparison, a protein‐based gel may also have advantages in digestibility and nutrition. PNP/HRGF emulsion gel was found to have superior digestibility as it achieved a 96.1% free fatty acid release over PNP‐only and HRGF‐only gel (88.8% and 70.9%, respectively). This was due to the smaller oil droplet size of the double‐network emulsion gel. The dense gel network in PNP/HRGF emulsion gel has also increased the stability of curcumin during digestion. The denser gel network and smaller droplet size subsequently contributed to improving the bioavailability of curcumin (Kong et al. 2024). On the other hand, modulation of digestion may also be easily achieved by altering gelator content. SPI/WPI emulsion gel induced by 0.3% CaCl_2_ was found to improve stability and have a sustained release of vitamin E (X. Zhang et al. 2022). The inclusion of a blended protein would supplement a functional protein that may be deficient in certain essential amino acids. The addition of potato protein with zein may deliver a more complete amino acid profile as outlined by Glusac et al. (2018). This was because potato protein has a high essential amino acid index while zein lacks lysine and tryptophan. Likewise, blending animal protein and plant protein would curate a more complete essential amino acid profile as the amino acid composition could be complementary. Blends such as dairy (limited by methionine + cysteine) with cereal (limited by lysine) or those with eggs could deliver more nutritionally appealing products (Day et al. 2022).

The formulation and creation of double protein network emulsion gels remain compelling. Compared with their single‐network counterparts, the creation of a double network improves WHC and freeze‐thaw stability. The modulation of these properties could be done by increasing the filled oil content and the degree of cross‐linkage through the use of gelator(s). This could be done without compromise on nutritional content and design constraints from biopolymer incompatibility. The active role of the dispersed oil phase in the gel matrix makes double protein network emulsion gels a viable candidate for creating high oil content products. Despite that, the field of creating binary protein blends that are both contributing to the gel network remains emerging. Efforts are still required to advance our understanding of the relationship and interactions between different protein sources, especially in the presence of oil.

## 6 Food application of Double‐Network Emulsion Gels

Food that is animal‐derived or traditionally created using animal ingredients may be replaced or enhanced using double‐network emulsion gels that are formulated using plant‐based or other ingredients. Researchers have developed both food components/ingredients and products such as animal fat, mayonnaise, and egg replacements. Double network emulsion gels may also act as functional carriers of bioactive ingredients with tunable release characteristics. Recently, double‐network emulsion gels have been developed into 3D‐printable ink. The added biopolymeric network is critical in creating an intermediate structure for high‐fidelity prints. This section seeks to outline tested applications of double‐network emulsion gel that have been reported in the literature. Table 4 shows various possible food applications of double‐network emulsion gels segregated by type, biopolymers used, and key qualities.

**TABLE 4:** Food application of double‐network emulsion gels.

Application	Type	Biopolymers	Qualities	Reference
Animal fat analog	PS/PS	MC, DKG	Thermal irreversible mechanical properties upon cooling Comparable hardness to pork fat achieved with 1:1 MC: DKG and the use of coconut oil.	(Jeong et al. 2023)
	PS/PS	Agar, SA	‐ Thermal irreversible mechanical properties upon heating ‐ Lighter (higher L*) than pork fat and more yellowish and slightly greenish than reddish pork fat. ‐ Comparable springiness but inferior hardness and chewiness to uncooked pork fat.	(Choi et al. 2023)
	PS/PS	CF, CD/DKG	‐ Thermal irreversible mechanical properties upon heating ‐ Emulsion gels with CF and CD or DKG showed G’ approaching pork belly fat/animal skin ‐ CF‐DKG gels showed shear stability, indicating suitability for extruded products.	(Tan and Phoon 2023)
	PS/PS	CS, SA	‐ Thermal irreversible mechanical properties upon heating ‐ Minimal cooking loss at 30% (v/v) oil	(Su et al. 2024)
Milk fat replacer	PS/PN	BCNF, SPI	‐ Substitution of milk fat with BCNF‐SPI emulsion gel could retard melting of ice cream ‐ Limited changes in sensory perception up to 60% substitution of milk fat with BCNG‐SPI emulsion gel ‐ Similar texture to full cream ice cream up to 90% substitution	(Gao et al. 2023)
Egg replacer	PS/PN	PP, β‐glucan	‐ Similar thermal gelation profile between PP‐β‐glucan emulsion gel (2.5–5% β‐glucan) and egg yolk ‐ Emulsion gels with 2.5% β‐glucan have similar springiness and chewiness to egg yolk but weaker hardness and resilience. ‐ Appearance may be modulated by the addition of β‐carotene	(S. S. Li et al. 2024)
	PS/PN	CPF, SPI, κC, starch, HAG	‐ A similar thermal gelation profile to liquid egg was achieved. ‐ Emulsion gel at 0.3% κC had similar hardness to egg omelet	(Lu et al. 2022)
Functional food	PS/PN	SPI, SBP	‐ Increased gel hardness from the addition of SBP ‐ SBP contributed to the controlled release of β‐carotene and riboflavin during digestion	(Feng et al. 2024; M. Zhang et al. 2022)
	PS/PN	WPI‐EGCG, HAG	‐ Increased probiotic survivability during digestion up to 0.2% HAG ‐ GDL‐induced gel created a more compact gel structure for increased survivability	(Qin et al. 2023)
	PS/PN	WPC‐XG, κC	‐ Increased thermal, storage, and digestion survivability for CA and K^+^‐induced gel ‐ The dense gel structure created by CA and K^+^‐induced gel increased survivability.	(Shen et al. 2024)
3D printing	PS/PN	PPI, κC	‐ Glycyrrhizic acid cross‐linking increased viscoelasticity and printability of ink ‐ 10% PPI with 0.3% Glycyrrhizic acid showed the highest fidelity in print	(Lin et al. 2024)
	PS/PN	PPI, SA, BCNF	‐ Shear thinning was observed for SA‐BCNF double‐network emulsion gel regardless of TGase addition. ‐ BNCF and TGase were shown to increase the fidelity of prints	(Wang et al. 2023)
	PS/PS	HS, κC	‐ κC content was critical to shape retention during printing ‐ Presence of HA and κC could limit heat aggregation of WPI during printing ‐ 0.5% κC and 3–6% HS/WPI were successful in printing custom Ready‐to‐eat custard cream.	(Cai et al. 2022)

Abbreviations: BCNF: bacterial cellulose nanofiber; CD: curdlan; CF: citrus fiber; CPF: chickpea flour; CS: corn starch; DKG: deacetylated konjac glucomannan; EGCG: (−)‐epigallocatechin‐3‐gallate conjugate; HAG: high‐acyl gellan gum; HS: hydroxypropylated starch; κC: κ‐carrageenan; MC: methylcellulose; PN: protein; PP: potato protein; PPI: pea protein isolate; PS: polysaccharide; SA: sodium alginate; SBP: sugar beet pectin; SPI: soy protein isolate; TGase: transglutaminase; WPC: whey protein concentrate; WPI: whey protein isolate; XG: xanthan gum.

### 6.1 Replacement of Animal‐Based Products

**FIGURE 5:**
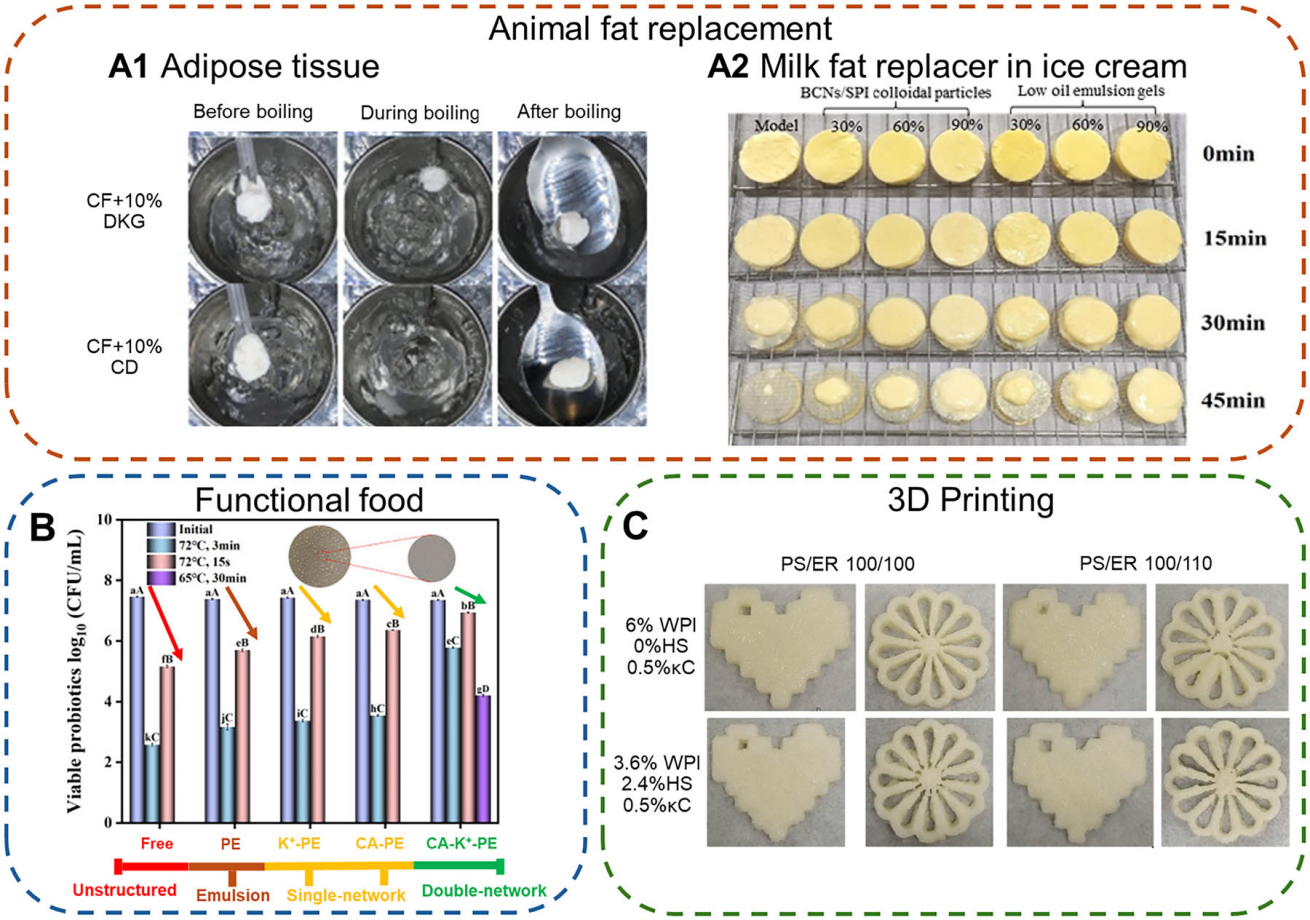
Potential applications of double‐network emulsion gels in food. (a1) Thermally irreversible double‐polysaccharide emulsion gel based on CF and DKG or CD. (a2) Retardation of melting in ice cream by replacing milk fat with low oil emulsion gel. *Source*: Reproduced with permission from Tan and Phoon (2023) and Gao et al. (2023), Copyright 2023, Elsevier and Copyright 2023, Elsevier. (b) Thermal protection of probiotic bacteria in protein‐polysaccharide emulsion gel. Free: free *L. acidophilus*. PE: *L. acidophilus* in WPC‐XG Pickering emulsion, K^+^‐PE: Pickering emulsion crosslinked by K^+^, CA‐PE: Pickering emulsion crosslinked by citric acid, CA‐K^+^‐PE: Pickering emulsion double crosslinked by citric acid and K^+^. *Source*: Reproduced with permission from Shen et al. (2024), Copyright 2024, Elsevier. (c) High‐fidelity printing of ready‐to‐eat custard at varying extruding rates (ER) and WPI and HS content. *Source*: Reproduced with permission from Cai et al. (2022), Copyright 2021, Elsevier.

Researchers had proposed to use double‐network emulsion gels as animal fat analogs in meat alternatives and fat replacers in frozen desserts. Animal fat tissue plays a critical role in the mouthfeel and other sensory attributes of meat during mastication. Retention of oil and texture after cooking is critical for these properties. Highly texturized and thermal‐irreversible animal fat analogs were created in research (Choi et al. 2023; Jeong et al. 2023; Su et al. 2024; Tan and Phoon 2023). The double‐polysaccharide regimes devised by Jeong et al. (2023) and Choi et al. (2023) were able to rectify the softening of MC (low temperature) and helix‐coil transition of agar (high temperature) using DKG and alginate, respectively. The use of citrus fiber and high‐set curdlan was able to maintain structural integrity after boiling in water (Tan and Phoon 2023) (Figure 5a1). On the other hand, the excellent freeze‐thaw stability created by the coexisting network demonstrated potential as a fat replacer in ice cream. Gao et al. (2023) devised a low‐oil Pickering emulsion gel using cellulose nanofiber and SPI that replaces cream in an ice cream formulation. A replacement ratio of up to 60% was found to have indistinguishable taste, aroma, and texture from a control full‐fat ice cream model. Retardation of melting rate (−18.23 to −61.14%) was also observed as fat replacement increased (30–90% replacement; Figure 5a2).

Double‐network emulsion gels could also be used as a replacement for egg components and whole‐egg products. Proteins from both the white and yolk of eggs are critical in the gelation of certain food items. S.S. Li et al. (2024) created an egg yolk analog using potato protein, β‐glucan, and olive oil. The emulsion gel formed by potato protein and β‐glucan formed a thermal‐irreversible gel through heating, much like that of egg yolk. The texture of the gel may be modulated through the addition of β‐glucan, as it could alter the formation of a protein gel. It was reported that the addition of 2.5% β‐glucan mimicked the chewiness and springiness of egg yolk. Lu et al. (2022) attempted to recreate whole eggs in an omelet‐like product with SPI, chickpea flour, starch, and κC or gellan gum. Samples created with 0.3% κC were found to mimic the texture of the control egg omelets. At 0.3% κC, the carrageenan and protein were able to interact via electrostatic interactions and form a synergistic network with void‐filling gelatinized starch granules. The creation of a double‐network emulsion gel may also improve the health aspect of mayonnaise with little compromise in texture and thixotropy (Yang et al. 2020). An egg yolk/KGM/alginate emulsion gel at 30% oil could achieve similar rheological properties to full‐fat mayonnaise (70% oil) (Yang et al. 2020).

### 6.2 Functional Foods

The simultaneous existence of two independent networks offers greater latitude in manipulating the release profile of bioactive ingredients. Lipid digestion may be delayed or hastened in double‐network emulsion gels (Feng et al. 2024; Kong et al. 2024; Liang et al. 2024; Xu et al. 2023; M. Zhang et al. 2022). For instance, an SPI‐SBP network was reported to restrict the contact between oil droplets and digestive enzymes, showing a slower, sustained release profile of β‐carotene that better preserves the molecule (Feng et al. 2024; M. Zhang et al. 2022). The bioaccessibility of β‐carotene in SPI/SBP emulsion gel (∼15%) after a simulated pectinase colonic model was greater than the emulsion‐only (∼10%) and SBP‐only emulsion gels (<5%). On the other hand, double protein emulsion gels may increase the release of free fatty acid and the bioavailability of curcumin due to smaller droplets and less aggregation (Kong et al. 2024). However, the bioaccessibility of lipophilic ingredients may also be impeded by highly viscous gums such as KGM. Using the standardized static digestion model, the addition of 0.4% KGM reduced the bioavailability of β‐carotene from 2.69% to 0.0028% (measured after intestinal digestion). This was due to the inability of bile salt and lipase to adhere to the KGM structure to access the dispersed droplets for digestion (Xu et al. 2023). A strong and dense gel network that was made possible by the creation of a double network may also translate to targeted release regimens. Double‐network emulsion gels were shown to be an effective method for delivering probiotic bacteria, with an increased rate of survivability (Qin et al. 2023; Shen et al. 2024). Probiotic bacteria were able to survive simulated static gastric digestion to have an increased release in the small intestine and colon. Again, these properties may still be dependent on the structure of the double network, as it was shown that an intermediate 0.2% gellan gum had the greatest protection at 85.3% of the initial loading in a study by Qin et al. (2023).

Aside from modulation of release, double‐network emulsion gels may increase the retention of photosensitive and thermal‐sensitive ingredients. The denser and stronger internal structure of double‐network emulsion gels provided enhanced stability when the sample was heated (Shen et al. 2024). Double‐network formation improved the survivability of *L. acidophilus* during pasteurization. Although a reduction in final viable cells was observed for double network emulsion gels (−1.58 log CFU/mL after 3 min at 72°C), the reduction is significantly smaller than that of free probiotics bacteria (−4.89 log CFU/mL after 3 min at 72°C) (Figure 5b) (Shen et al. 2024). Likewise, the dense network may impede the penetration of UV radiation within the emulsion gel (Miao et al. 2021; Yan et al. 2021). The absorption and scattering of light by the gel network and oil droplets may have also reduced exposure to radiation of the loaded bioactive ingredients (Miao et al. 2021; Yan et al. 2021). KGM was shown to increase the thickness of the interface and therefore impede the irradiation of β‐carotene (Xu et al. 2023). Lastly, the use of polyphenols such as tannic acid may also act as an antioxidant, preventing the oxidation of the loaded bioactive ingredient (Miao et al. 2021).

### 6.3 3D Printing

3D‐printed food garnered interest due to its customizability in appearance and morphology. The addition of a second structuring agent could achieve one of the following. Improvement of the final gel strength with no penalty on its printability (Y. Li et al. 2024; Lin et al. 2024; Wang et al. 2023). Improvement of flowability during extrusion at a lower primary structuring biopolymer concentration (Cai et al. 2022).

3D‐printing of rigid double‐network emulsion gel may involve modification of the formation process to suit varying gelation conditions of biopolymers (Lin et al. 2024; Wang et al. 2023). A 3D‐printable double‐network high internal phase emulsion gel was created by Wang et al. (2023). The researchers reported a two‐step approach to create the final emulsion gel. A TGase‐crosslinked pea protein network was the dominant gel network with dispersed bacterial cellulose nanofibers (BCNF)‐stabilized oil droplets and SA within the ink. The second alginate network was formed by immersion in CaCl_2_ solution for 24 h after the initial printing of the gel. The TGase‐crosslinked pea protein ink, with the help of BCNF entanglement, displayed desirable shear‐thinning and self‐supporting properties as an intermediate phase. Reinforcement of the gel structure was observed after CaCl_2_ immersion, where the hardness was increased by 1.86×. Thus, the sequential gelation of biopolymers is effective in the formation of a strong double‐network matrix postprinting with no or limited penalty in printability.

A network with multiple components was also seen to improve the printability of the ink to achieve high‐fidelity prints. In a WPI/hydroxypropylated starch (HS) and κC stabilized custard cream, the printability was increased as the two polysaccharides prevented excessive aggregation of WPI and increased viscosity (Cai et al. 2022). Synergistic interactions between HS and κC were observed at low HS content, with gelatinized HS providing stabilizing effects and κC providing a rigid gel network as it experienced coil‐helix transition when cooled. Sufficient self‐supporting properties could be achieved at 0.5% κC and 3–6% WPI/HS (2:3) with adequate preservation of details (Figure 5c). At high κC content (1–2%), the domination of κC helical interaction overwhelmingly dominated the gel structure at extrusion temperature (45°C), leading to misprints due to difficulty in extrusion (Cai et al. 2022). Thus, a balance of major and minor structuring polysaccharides and proteins is key to creating printable emulsion gels. In the case of the study, the balance of the two polysaccharides produced ready‐to‐eat emulsion gels with customizable appearance.

Several areas, such as the replacement of animal‐based food products, functional food, and 3D printing, have been identified as possible application areas. Through the adoption of a double‐network system, these emulsion gels were able to better target their desired properties, such as texture, thermal properties, bioavailability, and printability. Nonetheless, double‐polysaccharide and protein‐polysaccharide systems were the most prominent. Double‐protein systems were only used in limited cases. This indicates a potential area of development to create more appealing novel food products that could incorporate double‐network emulsion gels.

## 7 Outlook of Double‐Network Emulsion Gels

### 7.1 Industrial Adoption and Consumer Acceptance

Double‐network emulsion gels in food have been limited to research and have yet to have widespread industrial adoption. Emulsion gels, like their hydrogel counterparts, have several processing advantages over other texturization methods, such as extrusion. This includes not requiring specific machinery and lower energy requirements due to the lower thermal gelation conditions of food biopolymers (De Angelis et al. 2024; Kim et al. 2024). In the context of double‐network emulsion gels, the schematic of inducing gelation for the two networks would underpin its suitability for industrial‐scale adoption. A one‐pot or even a one‐step process would be favored over a two‐step method for minimizing handling and maximizing throughput in a double‐network structure (Sinad et al. 2023; Yin et al. 2023). A two‐step method that requires incubation in an ionic bath would be the most challenging for scalability and efficiency due to its dependency on the rate of diffusion and surface area‐to‐volume ratio. As contemporary studies focused on elucidating these gels’ formation mechanisms and properties on a lab scale, greater attention on processing could improve the appeal of double‐network emulsion gels.

The development of double‐network emulsion gel in foods has been focused on improving the consumer experience in a wide range of existing and novel food categories, including plant‐based meat, plant‐based cheese, confectionery, and condiments. Nonetheless, it remains unclear how its incorporation into food could affect the sensory perception of the entire product. Moreover, the perception of ultraprocessing has undermined the perceived healthiness of novel food products such as meat alternatives (Hässig et al. 2023). Many double‐network emulsion gels have also relied on allergenic protein sources such as dairy, egg, meat, and legume proteins for their network formations (Muthukumar et al. 2020). Overall, these issues have played a part in restricting the proliferation of novel products that could use double‐network emulsion gels. To improve the prospects of these products amidst these challenges, both transparency in materials used and processing done, are needed for more effective promotion of novel food products. Stricter compliance, self and otherwise, with regulatory standards would be needed to ensure safety and instill confidence in consumers, especially when novel materials are used. These novel materials may take the form of structural or chemical modifications of existing ingredients, such as polyphenol conjugation or new extracts of other food sources. On the other hand, less resistance may be seen in the adoption of double‐network emulsion gels as a delivery system for bioactive ingredients, as their benefits in functionality could be better communicated to consumers. Further research is warranted in double‐network emulsion gels to alleviate the multifaceted challenges currently faced in the development of novel food products. Further understanding of the synergistic effects between double‐network emulsion gels and their surrounding food matrix could better promote the use of and adoption of double‐network emulsion gel by the industry and consumers.

### 7.2 Future Research

The use of common gelling agents seen in current research is a natural extension to the established single network emulsion gels. Future investigations may be dedicated to using underutilized plant and animal biopolymers will be indispensable in creating novel structures. Research into underutilized protein sources has yielded dividends when applied to simpler gel designs. Protein extracts from oilseeds, pseudocereals, and marine organisms were seen in literature to create hydrogels and, in certain cases, emulsion gels. Canola protein extracts from spent canola meal have shown promising emulsifying and gelling properties in research (Lerch et al. 2024; Tan et al. 2014). Recent advances in protein preparations have also enabled cold‐set gelation of canola protein that was previously heat‐induced. Lerch et al. (2024) determined that alkaline extraction at pH 12 was a prerequisite for forming a 3D CaCl_2_ cross‐linked gel matrix. Pseudocereals such as quinoa proteins have shown favorable Pickering properties and interactions with a myofibrillar protein gel with increased WHC and gel strength up to 7.5% (w/w) Pickering droplet addition (Cen et al. 2022). The synergy between oyster protein and κC was identified by Jiang et al. (2022). The addition of κC to oyster protein increased the proportion of β‐sheets and created more orderly cross‐linkages as a result. All in all, the use of underutilized biopolymers and their combinations with other biopolymers will be instrumental in identifying novel uses for current agricultural products and reducing wastage. Food upcycling may also be achieved as by‐products and surplus may be used in more value‐adding processes and products (Aschemann‐Witzel et al. 2023).

Currently, mixed results were seen in the creation of double‐protein systems with limited experiments on how these systems would perform as food. An expanded investigation into protein‐based systems could maintain the healthy perception of plant‐based food while improving current challenges in inferior physical properties. Increasing protein fraction utilization for supplementary networks or functions may aid the creation of “clean label” foods by reducing the ingredient list. (Aschemann‐Witzel et al. 2021). Expanding the use of prolamins from familiar protein sources such as corn, wheat, and barley in creating double network emulsion gel may also unlock better prospects for developing a protein‐based product. Prolamin‐based hydrogels have shown excellent strength and were found to have synergy when used with other proteins to form complexes (Jia et al. 2020; J. J. Wang et al. 2019; Zhao et al. 2023). Blending animal protein with plant protein in developing double‐network emulsion gels may serve as a short‐term intermediary to reduce our consumption of animal‐sourced foods. Consumers were shown to favor animal‐like products over purely plant‐based products and that substitution of animal protein with plant does not immediately impact sensory favorability (Grasso and Goksen 2023; Sogari et al. 2023). Thus, expanding the use of prolamins with the inclusion of other protein sources, including blends of both animal and plant proteins, may hold the key to unlocking the full potential of a protein‐based system. In such a format, emulsifying capacity, protein profile, and gel properties may be accurately optimized by different protein components.

Unique properties of individual biopolymers (such as gel–sol transition temperature) may be better exploited to recreate animal‐based products. Double‐network emulsion gels are created as food analogs to tailor for varying temperature and pH responses of food gels. Food gels are required to sustain temperatures from freezing to high‐temperature cooking and acidity from weak during fermentation to chemical digestion at low pH. Difficulties had been observed in creating plant‐based food analogs as direct substitution of structuring agent yielded poor results. In food like cheeses, a casein emulsion gel of milk fat that has low pH and thermal reversibility, current analogs struggle to recreate key melting and stretching properties. The creation of emulsion gels based on multiple structuring agents may help target these thermal and mechanical properties. Thus, further exploration of methods to utilize the unique properties of individual biopolymers to form a synergistic network could assist in the delivery of a greater variety of food properties.

## 8 Conclusion

Double‐network emulsion gels have emerged as a novel method to structure emulsions in food. The combination of the food proteins and polysaccharides yielded enhanced and additional properties greater and unseen in single network emulsion gels that could be modulated.

In a protein‐polysaccharide network, polysaccharide enhanced WHC and texture due to its hydrophilic and void‐filling nature. Nonetheless, incompatibility was observed in systems with high polysaccharide content as phase separation became apparent. Tunable thermal properties may be achieved in double polysaccharide gels when a thermal reversible and irreversible blend of polysaccharides is used. Whether the dispersed droplets are active or inactive in double‐polysaccharide gels may implicate the gel's suitability at high oil concentrations. This may be modified depending on the selection of polysaccharides. As protein acts as both the emulsifier and structuring agent, oil droplets are typically active in double‐protein systems. Despite that, the nature of the gel network is still dependent on the interactions between the two proteins and their respective gelator, as differences between the proteins in gelation conditions may cause malformation of the double network.

Various applications of double‐network emulsion gels including, food analogs, functional food, and 3D printing have been reported in the literature. The existence of two networks is more versatile in tailoring the performance of the emulsion gel. Several areas have also been identified for further research, including the exploration of underutilized biopolymers to create novel structures, the use of various protein types and fractions to create protein‐based emulsion gels, and the use of a double‐network system to target specific culinary properties of food. The double‐network emulsion gel has been shown to be promising in potentially solving key challenges in creating texturized and functional foods.

## Author Contributions

**Canice Chun‐Yin Yiu**: conceptualization, investigation, writing–original draft, visualization, methodology, data curation, and formal analysis. **Yong Wang**: conceptualization, writing–review and editing, supervision, formal analysis, and project administration. **Cordelia Selomulya**: conceptualization, supervision, funding acquisition, writing–review and editing, resources, and project administration.

## Conflicts of Interest

The authors declare no conflicts of interest.

## Data Availability

No data was generated.

## References

[crf370201-bib-0001] Abbaszadeh, A. , W. MacNaughtan , G. Sworn , and T. J. Foster 2016. “New Insights Into Xanthan Synergistic Interactions With Konjac Glucomannan: A Novel Interaction Mechanism Proposal.” Carbohydrate Polymers 144: 168–177. 10.1016/j.carbpol.2016.02.026. 27083806

[crf370201-bib-0002] Amici, E. , A. H. Clark , V. Normand , and N. B. Johnson 2002. “Interpenetrating Network Formation in Agarose–Kappa‐Carrageenan Gel Composites.” Biomacromolecules 3, no. 3: 466–474. 10.1021/bm010157z. 12005516

[crf370201-bib-0003] Aschemann‐Witzel, J. , D. Asioli , M. Banovic , M. A. Perito , A. O. Peschel , and V. Stancu 2023. “Defining Upcycled Food: The Dual Role of Upcycling in Reducing Food Loss and Waste.” Trends in Food Science & Technology 132: 132–137. 10.1016/j.tifs.2023.01.001.

[crf370201-bib-0004] Aschemann‐Witzel, J. , R. F. Gantriis , P. Fraga , and F. J. A. Perez‐Cueto 2021. “Plant‐Based Food and Protein Trend From a Business Perspective: Markets, Consumers, and the Challenges and Opportunities in the Future.” Critical Reviews in Food Science and Nutrition 61, no. 18: 3119–3128. 10.1080/10408398.2020.1793730. 32654499

[crf370201-bib-0005] Aschemann‐Witzel, J. , and A. O. Peschel 2019. “Consumer Perception of Plant‐Based Proteins: The Value of Source Transparency for Alternative Protein Ingredients.” Food Hydrocolloids 96: 20–28. 10.1016/j.foodhyd.2019.05.006.

[crf370201-bib-0006] Cai, J. , D. Zhang , and F. Xie 2024. “The Role of Alginate in Starch Nanocrystals‐Stabilized Pickering Emulsions: From Physical Stability and Microstructure to Rheology Behavior.” Food Chemistry 431: 137017. 10.1016/j.foodchem.2023.137017. 37562336

[crf370201-bib-0007] Cai, Q. Y. , Y. L. Zhong , M. L. Xu , Q. R. Huang , and X. X. Lu 2022. “3D printed High Oil Custard Cream: Effects of Whey Protein Isolate, Hydroxypropylated Starch and Carrageenan on Physicochemical Properties and Printing Performance.” Lwt‐Food Science and Technology 156: 113039. 10.1016/j.lwt.2021.113039.

[crf370201-bib-0008] Cen, K. , X. Yu , C. Gao , Y. Yang , X. Tang , and X. Feng 2022. “Effects of Quinoa Protein Pickering Emulsion on the Properties, Structure and Intermolecular Interactions of Myofibrillar Protein Gel.” Food Chemistry 394: 133456. 10.1016/j.foodchem.2022.133456. 35717909

[crf370201-bib-0009] Chen, H. , J. Gan , A. Ji , S. Song , and L. Yin 2019. “Development of Double Network Gels Based on Soy Protein Isolate and Sugar Beet Pectin Induced by Thermal Treatment and Laccase Catalysis.” Food Chemistry 292: 188–196. 10.1016/j.foodchem.2019.04.059. 31054664

[crf370201-bib-0010] Chen, Q. , H. Chen , L. Zhu , and J. Zheng 2015. “Fundamentals of Double Network Hydrogels.” J Mater Chem B 3, no. 18: 3654–3676. 10.1039/c5tb00123d. 32262840

[crf370201-bib-0011] Cheng, Y. , A. Ye , and H. Singh 2024. “Characterizations of Emulsion Gel Formed with the Mixture of Whey and Soy Protein and Its Protein Digestion Under In Vitro Gastric Conditions.” Curr Res Food Sci 8: 100674. 10.1016/j.crfs.2023.100674. 38283161 PMC10818200

[crf370201-bib-0012] Choi, M. , H. W. Choi , H. Kim , J. Hahn , and Y. J. Choi 2023. “Mimicking Animal Adipose Tissue Using a Hybrid Network‐Based Solid‐Emulsion Gel With Soy Protein Isolate, Agar, and Alginate.” Food Hydrocolloids 145: 109043. 10.1016/j.foodhyd.2023.109043.

[crf370201-bib-0013] Cornet, S. H. V. , A. J. van der Goot , and R. G. M. van der Sman 2020. “Effect of Mechanical Interaction on the Hydration of Mixed Soy Protein and Gluten Gels.” Curr Res Food Sci 3: 134–145. 10.1016/j.crfs.2020.03.007. 32914129 PMC7473356

[crf370201-bib-0014] Day, L. , J. A. Cakebread , and S. M. Loveday 2022. “Food Proteins from Animals and Plants: Differences in the Nutritional and Functional Properties.” Trends in Food Science & Technology 119: 428–442. 10.1016/j.tifs.2021.12.020.

[crf370201-bib-0015] De Angelis, D. , A. J. van der Goot , A. Pasqualone , and C. Summo 2024. “Advancements in Texturization Processes for the Development of Plant‐Based Meat Analogs: a Review.” Current Opinion in Food Science 58: 101192. 10.1016/j.cofs.2024.101192.

[crf370201-bib-0016] Dickinson, E. 2003. “Hydrocolloids at Interfaces and the Influence on the Properties of Dispersed Systems.” Food Hydrocolloids 17, no. 1: 25–39. 10.1016/s0268-005x(01)00120-5.

[crf370201-bib-0017] Dickinson, E. 2012. “Emulsion Gels: The Structuring of Soft Solids with Protein‐Stabilized Oil Droplets.” Food Hydrocolloids 28, no. 1: 224–241. 10.1016/j.foodhyd.2011.12.017.

[crf370201-bib-0018] Dreher, J. , C. Blach , N. Terjung , M. Gibis , and J. Weiss 2020. “Formation and Characterization of Plant‐based Emulsified and Crosslinked Fat Crystal Networks to Mimic Animal Fat Tissue.” Journal of Food Science 85, no. 2: 421–431. 10.1111/1750-3841.14993. 31943214

[crf370201-bib-0019] Du, M. , W. Lu , Y. Zhang , A. Mata , and Y. Fang 2021. “Natural Polymer‐sourced Interpenetrating Network Hydrogels: Fabrication, Properties, Mechanism and Food Applications.” Trends in Food Science & Technology 116: 342–356. 10.1016/j.tifs.2021.07.031.

[crf370201-bib-0020] Du, M. , Y. Zhao , Y. Zhang , S. Sun , and Y. Fang 2022. “Fabrication of Agarose/Fish Gelatin Double‐Network Hydrogels with High Strength and Toughness for the Development of Artificial Beef Tendons.” Food Funct 13, no. 13: 6975–6986. 10.1039/d2fo00754a. 35678706

[crf370201-bib-0021] Du, M. J. , Y. Zhang , Y. G. Zhao , and Y. P. Fang 2023. “Agarose/Konjac Glucomannan Double Network Hydrogels to Mimic the Texture of Beef Tripe.” Food Hydrocolloids 135: 108173. 10.1016/j.foodhyd.2022.108173.

[crf370201-bib-0022] Feng, L. , X. Jia , and L. Yin 2024. “Role of Pectin in the Delivery of Beta‐Carotene Embedded in Interpenetrating Emulsion‐Filled Gels Made With Soy Protein Isolate.” Food Chemistry 446: 138797. 10.1016/j.foodchem.2024.138797. 38442678

[crf370201-bib-0023] Gao, Y. , D. Lin , H. Peng , R. Zhang , B. Zhang , and X. Yang 2023. “Low Oil Pickering Emulsion Gels Stabilized by Bacterial Cellulose Nanofiber/Soybean Protein Isolate: An Excellent Fat Replacer for Ice Cream.” International Journal of Biological Macromolecules 247: 125623. 10.1016/j.ijbiomac.2023.125623. 37392915

[crf370201-bib-0024] Ghiraldi, M. , B. G. Franco , I. C. F. Moraes , and S. C. Pinho 2021. “Emulsion‐Filled Pectin Gels for Vehiculation of Vitamins D3 and B12: From Structuring to the Development of Enriched Vegan Gummy Candies.” ACS Food Science & Technology 1, no. 10: 1945–1952. 10.1021/acsfoodscitech.1c00271.

[crf370201-bib-0025] Glusac, J. , I. Davidesko‐Vardi , S. Isaschar‐Ovdat , B. Kukavica , and A. Fishman 2018. “Gel‐Like Emulsions Stabilized by Tyrosinase‐Crosslinked Potato and Zein Proteins.” Food Hydrocolloids 82: 53–63. 10.1016/j.foodhyd.2018.03.046.

[crf370201-bib-0026] Grasso, S. , and G. Goksen 2023. “The Best of both Worlds? Challenges and Opportunities in the Development of Hybrid Meat Products from the Last 3 Years.” Lwt 173: 114235. 10.1016/j.lwt.2022.114235.

[crf370201-bib-0027] Grinberg, V. Y. , and V. B. Tolstoguzov 1997. “Thermodynamic Incompatibility of Proteins and Polysaccharides in Solutions.” Food Hydrocolloids 11, no. 2: 145–158. 10.1016/s0268-005x(97)80022-7.

[crf370201-bib-0028] Gu, Z. , K. Huang , Y. Luo , et al. 2018. “Double Network Hydrogel for Tissue Engineering.” Wiley Interdisciplinary Reviews: Nanomedicine and Nanobiotechnology 10, no. 6: e1520. 10.1002/wnan.1520. 29664220

[crf370201-bib-0029] Hässig, A. , C. Hartmann , L. Sanchez‐Siles , and M. Siegrist 2023. “Perceived Degree of Food Processing as a Cue for Perceived Healthiness: The NOVA System Mirrors Consumers' Perceptions.” Food Quality and Preference 110: 104944. 10.1016/j.foodqual.2023.104944.

[crf370201-bib-0030] Hou, W. , J. Long , Y. Hua , et al. 2022. “Formation and Characterization of Solid Fat Mimetic Based on Pea Protein Isolate/Polysaccharide Emulsion Gels.” Frontiers in Nutrition 9: 1053469. 10.3389/fnut.2022.1053469. 36438737 PMC9684638

[crf370201-bib-0031] Hu, X. , P. Karthik , and J. Chen 2021. “Enhanced Oral Oil Release and Mouthfeel Perception of Starch Emulsion Gels.” Food Research International 144: 110356. 10.1016/j.foodres.2021.110356. 34053549

[crf370201-bib-0032] Jeong, H. , J. Lee , Y.‐J. Jo , and M.‐J. Choi 2023. “Thermo‐Irreversible Emulsion Gels Based on Deacetylated Konjac Glucomannan and Methylcellulose as Animal Fat Analogs.” Food Hydrocolloids 137: 108407. 10.1016/j.foodhyd.2022.108407.

[crf370201-bib-0033] Jia, F. , J. J. Wang , Y. X. Wang , et al. 2020. “Fabrication and Characterization of Wheat Gliadin Hydrogels With High Strength and Toughness.” Journal of Cereal Science 95: 103038. 10.1016/j.jcs.2020.103038.

[crf370201-bib-0034] Jiang, Q. , P. Li , M. Ji , et al. 2022. “Synergetic Effects of Water‐Soluble Polysaccharides for Intensifying Performances of Oleogels Fabricated by Oil‐Absorbing Cryogels.” Food Chemistry 372: 131357. 10.1016/j.foodchem.2021.131357. 34655833

[crf370201-bib-0035] Jiang, Y. , L. Liu , B. Wang , et al. 2019. “Polysaccharide‐Based Edible Emulsion Gel Stabilized by Regenerated Cellulose.” Food Hydrocolloids 91: 232–237. 10.1016/j.foodhyd.2019.01.028.

[crf370201-bib-0036] Jose, J. , L. Pouvreau , and A. H. Martin 2016. “Mixing Whey and Soy Proteins: Consequences for the Gel Mechanical Response and Water Holding.” Food Hydrocolloids 60: 216–224. 10.1016/j.foodhyd.2016.03.031.

[crf370201-bib-0037] Kim, W. , C. C. Yiu , Y. Wang , W. Zhou , and C. Selomulya 2024. “Toward Diverse Plant Proteins for Food Innovation.” Adv Sci (Weinh) e2408150. 10.1002/advs.202408150. 39119828 PMC11892504

[crf370201-bib-0038] Kobayashi, K. , C.‐I. Huang , and T. P. Lodge 1999. “Thermoreversible Gelation of Aqueous Methylcellulose Solutions.” Macromolecules 32, no. 21: 7070–7077. 10.1021/ma990242n.

[crf370201-bib-0039] Kong, Z. , Z. Li , L. Zhang , et al. 2024. “Development of Pea Protein Nanoparticle/Hydrolyzed Rice Glutelin Fibril Emulsion Gels for Encapsulation of Curcumin.” International Journal of Biological Macromolecules 276, no. Pt 1: 133640. 10.1016/j.ijbiomac.2024.133640. 38969047

[crf370201-bib-0040] Lambrecht, M. A. , I. Rombouts , B. De Ketelaere , and J. A. Delcour 2017. “Prediction of Heat‐Induced Polymerization of Different Globular Food Proteins in Mixtures With Wheat Gluten.” Food Chemistry 221: 1158–1167. 10.1016/j.foodchem.2016.11.043. 27979074

[crf370201-bib-0041] Lerch, N. L. , A. Vahedifar , J. Weiss , and J. P. Wu 2024. “Cold Gelation of Canola Protein Isolate and Canola Protein Hydrolysates.” Food Hydrocolloids 152: 109840. 10.1016/j.foodhyd.2024.109840.

[crf370201-bib-0042] Li, H. , D. Hao , J. Fan , et al. 2016. “A Robust Double‐network Hydrogel with Under Sea Water Superoleophobicity Fabricated via One‐Pot, One‐Step Reaction.” J Mater Chem B 4, no. 27: 4662–4666. 10.1039/c6tb00818f. 32263237

[crf370201-bib-0043] Li, J. , R. Niu , Q. Zhu , et al. 2023. “Nanostarch‐enhanced 3D Printability of Carrageenan Emulsion Gel for High‐fidelity and Nutrition‐fortified Fish Fat Mimics.” Food Hydrocolloids 145: 109099. 10.1016/j.foodhyd.2023.109099.

[crf370201-bib-0044] Li, P. Y. , C. Guo , X. F. Li , et al. 2021. “Preparation and Structural Characteristics of Composite Alginate/Casein Emulsion Gels: A Microscopy and Rheology Study.” Food Hydrocolloids 118: 106792. 10.1016/j.foodhyd.2021.106792.

[crf370201-bib-0045] Li, S. , G. Chen , X. Shi , C. Ma , and F. Liu 2022. “Comparative Study of Heat‐ and Enzyme‐Induced Emulsion Gels Formed by Gelatin and Whey Protein Isolate: Physical Properties and Formation Mechanism.” Gels 8, no. 4: 212. 10.3390/gels8040212. 35448113 PMC9027002

[crf370201-bib-0046] Li, S. S. , M. N. Luo , D. Wannasin , et al. 2024. “Exploring the Potential of Plant‐based Emulsion Gels Enriched With β‐Glucan and Potato Protein as Egg Yolk Alternatives.” Food Hydrocolloids 148: 109511. 10.1016/j.foodhyd.2023.109511.

[crf370201-bib-0047] Li, X. , X. Chen , and H. Cheng 2024. “Impact of Kappa‐Carrageenan on the Cold‐Set Pea Protein Isolate Emulsion‐Filled Gels: Mechanical Property, Microstructure, and in Vitro Digestive Behavior.” Foods 13, no. 3: 483. 10.3390/foods13030483. 38338618 PMC10855759

[crf370201-bib-0048] Li, Y. , J. Wang , R. Ying , M. Huang , and K. Hayat 2024. “Protein‐stabilized Pickering Emulsion Interacting With Inulin, Xanthan Gum and Chitosan: Rheological Behavior and 3D Printing.” Carbohydrate Polymers 326: 121658. 10.1016/j.carbpol.2023.121658. 38142086

[crf370201-bib-0049] Liang, B. , S. Feng , X. Zhang , et al. 2024. “Physicochemical Properties and in Vitro Digestion Behavior of Emulsion Micro‐gels Stabilized by Kappa‐Carrageenan and Whey Protein: Effects of Sodium Alginate Addition.” International Journal of Biological Macromolecules 271, no. Pt 1: 132512. 10.1016/j.ijbiomac.2024.132512. 38795879

[crf370201-bib-0050] Liang, X. P. , C. C. Ma , X. J. Yan , et al. 2020. “Structure, Rheology and Functionality of Whey Protein Emulsion Gels: Effects of Double Cross‐linking With Transglutaminase and Calcium Ions.” Food Hydrocolloids 102: 105569. 10.1016/j.foodhyd.2019.105569.

[crf370201-bib-0051] Lin, D. Q. , A. L. Kelly , and S. Miao 2020. “Preparation, Structure‐property Relationships and Applications of Different Emulsion Gels: Bulk Emulsion Gels, Emulsion Gel Particles, and Fluid Emulsion Gels.” Trends in Food Science & Technology 102: 123–137. 10.1016/j.tifs.2020.05.024.

[crf370201-bib-0052] Lin, Q. Z. , M. S. Shang , X. J. Li , et al. 2024. “Rheology and 3D Printing Characteristics of Heat‐Inducible Pea Protein‐Carrageenan‐Glycyrrhizic Acid Emulsions as Edible Inks.” Food Hydrocolloids 147: 109347. 10.1016/j.foodhyd.2023.109347.

[crf370201-bib-0053] Lin, Y. C. , H. Du , Y. Roos , and S. Miao 2023. “Binary Complexes of Whey Protein Fibers/Isolates and Fish Gelatins for Emulsion Stabilization.” Food Hydrocolloids 143: 108880. 10.1016/j.foodhyd.2023.108880.

[crf370201-bib-0054] Line, V. L. S. , G. E. Remondetto , and M. Subirade 2005. “Cold Gelation of β‐Lactoglobulin Oil‐in‐Water Emulsions.” Food Hydrocolloids 19, no. 2: 269–278. 10.1016/j.foodhyd.2004.06.004.

[crf370201-bib-0055] Lü, T. , Y. Wu , Y. Tao , D. Zhang , D. Qi , and H. Zhao 2020. “Facile Synthesis of Octyl‐Modified Alginate for Oil‐water Emulsification.” Colloid and Polymer Science 298, no. 12: 1637–1648. 10.1007/s00396-020-04745-x.

[crf370201-bib-0056] Lu, Y. , L. Mao , Z. Hou , S. Miao , and Y. Gao 2019. “Development of Emulsion Gels for the Delivery of Functional Food Ingredients: From Structure to Functionality.” Food Engineering Reviews 11, no. 4: 245–258. 10.1007/s12393-019-09194-z.

[crf370201-bib-0057] Lu, Z. , P. R. Lee , and H. S. Yang 2022. “Chickpea Flour and Soy Protein Isolate Interacted With κ‐Carrageenan via Electrostatic Interactions to Form Egg Omelets Analogue.” Food Hydrocolloids 130: 107691. 10.1016/j.foodhyd.2022.107691.

[crf370201-bib-0058] Mattice, K. D. , and A. G. Marangoni 2021. “Physical Properties of Zein Networks Treated with Microbial Transglutaminase.” Food Chemistry 338: 128010. 10.1016/j.foodchem.2020.128010. 32932084

[crf370201-bib-0059] McClements, D. J. 2024. “Composite Hydrogels Assembled from Food‐Grade Biopolymers: Fabrication, Properties, and Applications.” Advances in Colloid and Interface Science 332: 103278. 10.1016/j.cis.2024.103278. 39153416

[crf370201-bib-0060] McClements, D. J. , and S. M. Jafari 2018. “Improving Emulsion Formation, Stability and Performance Using Mixed Emulsifiers: A Review.” Advances in Colloid and Interface Science 251: 55–79. 10.1016/j.cis.2017.12.001. 29248154

[crf370201-bib-0061] Miao, J. Y. , N. Xu , C. Cheng , et al. 2021. “Fabrication of Polysaccharide‐Based High Internal Phase Emulsion Gels: Enhancement of Curcumin Stability and Bioaccessibility.” Food Hydrocolloids 117: 106679. 10.1016/j.foodhyd.2021.106679.

[crf370201-bib-0062] Muthukumar, J. , P. Selvasekaran , M. Lokanadham , and R. Chidambaram 2020. “Food and Food Products Associated with Food Allergy and Food Intolerance—An Overview.” Food Research International 138, no. Pt B: 109780. 10.1016/j.foodres.2020.109780. 33288166

[crf370201-bib-0063] Nakauma, M. , T. Funami , S. Noda , et al. 2008. “Comparison of Sugar Beet Pectin, Soybean Soluble Polysaccharide, and Gum Arabic as Food Emulsifiers. 1. Effect of Concentration, pH, and Salts on the Emulsifying Properties.” Food Hydrocolloids 22, no. 7: 1254–1267. 10.1016/j.foodhyd.2007.09.004.

[crf370201-bib-0064] Nicolai, T. 2019. “Gelation of Food Protein‐Protein Mixtures.” Advances in Colloid and Interface Science 270: 147–164. 10.1016/j.cis.2019.06.006. 31229885

[crf370201-bib-0065] Nishinari, K. 2021. “Gelling Properties.” In Food Hydrocolloids. 10.1007/978-981-16-0320-4_4. PMC7467918 32895590

[crf370201-bib-0066] Oliver, L. , E. Scholten , and G. A. van Aken 2015. “Effect of Fat Hardness on Large Deformation Rheology of Emulsion‐Filled Gels.” Food Hydrocolloids 43: 299–310. 10.1016/j.foodhyd.2014.05.031.

[crf370201-bib-0067] Qin, X. , Q. Bo , P. Qin , S. Wang , and K. Liu 2023. “Fabrication of WPI‐EGCG Covalent Conjugates/Gellangum Double Network Emulsion Gels by Duo‐Induction of GDL and CaCl(2) for Colon‐Controlled *Lactobacillus plantarum* Delivery.” Food Chemistry 404, no. Pt A: 134513. 10.1016/j.foodchem.2022.134513. 36240556

[crf370201-bib-0068] Ren, Y. , L. Huang , Y. Zhang , et al. 2022. “Application of Emulsion Gels as Fat Substitutes in Meat Products.” Foods 11, no. 13:. 10.3390/foods11131950. PMC9265990 35804763

[crf370201-bib-0069] Sarkar, A. , and E. Dickinson 2020. “Sustainable Food‐Grade Pickering Emulsions Stabilized by Plant‐based Particles.” Current Opinion in Colloid & Interface Science 49: 69–81. 10.1016/j.cocis.2020.04.004.

[crf370201-bib-0070] Schmidt, J. M. , H. Damgaard , M. Greve‐Poulsen , A. V. Sunds , L. B. Larsen , and M. Hammershoj 2019. “Gel Properties of Potato Protein and the Isolated Fractions of Patatins and Protease Inhibitors—Impact of Drying Method, Protein Concentration, pH and Ionic Strength.” Food Hydrocolloids 96: 246–258. 10.1016/j.foodhyd.2019.05.022.

[crf370201-bib-0071] Schmitt, C. , J. V. C. Silva , L. Amagliani , C. Chassenieux , and T. Nicolai 2019. “Heat‐Induced and Acid‐induced Gelation of Dairy/Plant Protein Dispersions and Emulsions.” Current Opinion in Food Science 27: 43–48. 10.1016/j.cofs.2019.05.002.

[crf370201-bib-0072] Schroen, K. , X. Shen , F. I. Hasyyati , S. Deshpande , and J. van der Gucht 2024. “From Theoretical Aspects to Practical Food Pickering Emulsions: Formation, Stabilization, and Complexities Linked to the Use of Colloidal Food Particles.” Advances in Colloid and Interface Science 334: 103321. 10.1016/j.cis.2024.103321. 39486347

[crf370201-bib-0073] Shen, J. , Y. Chen , X. Li , X. Zhou , and Y. Ding 2024. “Enhanced Probiotic Viability in Innovative Double‐network Emulsion Gels: Synergistic Effects of the Whey Protein Concentrate‐Xanthan Gum Complex and Kappa‐Carrageenan.” International Journal of Biological Macromolecules 270, no. Pt 1: 131758. 10.1016/j.ijbiomac.2024.131758. 38714282

[crf370201-bib-0074] Silva, J. V. C. , B. Jacquette , L. Amagliani , C. Schmitt , T. Nicolai , and C. Chassenieux 2019. “Heat‐Induced Gelation of Micellar Casein/Plant Protein Oil‐in‐Water Emulsions.” Colloids and Surfaces a‐Physicochemical and Engineering Aspects 569: 85–92. 10.1016/j.colsurfa.2019.01.065.

[crf370201-bib-0075] Sinad, K. V. G. , R. C. Ebubechukwu , and C. K. Chu 2023. “Recent Advances in Double Network Hydrogels Based on Naturally‐Derived Polymers: Synthesis, Properties, and Biological Applications.” J Mater Chem B 11, no. 48: 11460–11482. 10.1039/d3tb00773a. 38047404

[crf370201-bib-0076] Sogari, G. , V. Caputo , A. Joshua Petterson , C. Mora , and F. Boukid 2023. “A Sensory Study on Consumer Valuation for Plant‐Based Meat Alternatives: What Is Liked and Disliked the Most?” Food Research International 169: 112813. 10.1016/j.foodres.2023.112813. 37254388

[crf370201-bib-0077] Su, C. , Y. Feng , J. Ye , et al. 2018. “Effect of Sodium Alginate on the Stability of Natural Soybean Oil Body Emulsions.” RSC Advances 8, no. 9: 4731–4741. 10.1039/c7ra09375f. 35539521 PMC9077793

[crf370201-bib-0078] Su, C. Y. , D. Li , L. J. Wang , and Y. Wang 2024. “Development of Corn Starch‐sodium Alginate Emulsion Gels as Animal Fat Substitute: Effect of Oil Concentration.” Food Hydrocolloids 157: 110439. 10.1016/j.foodhyd.2024.110439.

[crf370201-bib-0079] Tan, S. H. , R. J. Mailer , C. L. Blanchard , and S. O. Agboola 2014. “Emulsifying Properties of Proteins Extracted from Australian Canola Meal.” Lwt‐Food Science and Technology 57, no. 1: 376–382. 10.1016/j.lwt.2013.12.040.

[crf370201-bib-0080] Tan, Z. L. A. , and P. Y. Phoon 2023. “Developing Concentrated Emulsion Gel Hybrids Structured by Natural Food Fibres.” Food Hydrocolloids 145: 109037. 10.1016/j.foodhyd.2023.109037.

[crf370201-bib-0081] Tao, H. , L. Guo , Z. Qin , et al. 2022. “Textural Characteristics of Mixed Gels Improved by Structural Recombination and the Formation of Hydrogen Bonds Between Curdlan and Carrageenan.” Food Hydrocolloids 129: 107678. 10.1016/j.foodhyd.2022.107678.

[crf370201-bib-0082] Tobin, J. T. , S. M. Fitzsimons , V. Chaurin , A. L. Kelly , and M. A. Fenelon 2012. “Thermodynamic Incompatibility Between Denatured Whey Protein and Konjac Glucomannan.” Food Hydrocolloids 27, no. 1: 201–207. 10.1016/j.foodhyd.2011.07.004.

[crf370201-bib-0083] Valle, C. , F. Echeverría , V. Chávez , R. Valenzuela , and A. Bustamante 2024. “Deep‐Frying Impact on Food and Oil Chemical Composition: Strategies to Reduce Oil Absorption in the Final Product.” Food Safety and Health 2, no. 4: 414–428. 10.1002/fsh3.12056.

[crf370201-bib-0084] van Oosten, A. S. G. , X. Chen , L. Chin , et al. 2019. “Emergence of Tissue‐Like Mechanics from Fibrous Networks Confined by Close‐Packed Cells.” Nature 573, no. 7772: 96–101. 10.1038/s41586-019-1516-5. 31462779

[crf370201-bib-0085] Wang, J. J. , Y. X. Wang , Q. Y. Wang , J. Q. Yang , S. Q. Hu , and L. Y. Chen 2019. “Mechanically Strong and Highly Tough Prolamin Protein Hydrogels Designed from Double‐Cross‐Linked Assembled Networks.” ACS Applied Polymer Materials 1, no. 6: 1272–1279. 10.1021/acsapm.9b00066.

[crf370201-bib-0086] Wang, S. , Z. Wu , L. Jia , et al. 2024. “Soybean Protein Isolate‐sodium Alginate Double Network Emulsion Gels: Mechanism of Formation and Improved Freeze‐thaw Stability.” International Journal of Biological Macromolecules 274, no. Pt 1: 133296. 10.1016/j.ijbiomac.2024.133296. 38914399

[crf370201-bib-0087] Wang, X. F. , K. Y. Luo , S. T. Liu , B. Adhikari , and J. Chen 2019. “Improvement of Gelation Properties of Soy Protein Isolate Emulsion Induced by Calcium Cooperated with Magnesium.” Journal of Food Engineering 244: 32–39. 10.1016/j.jfoodeng.2018.09.025.

[crf370201-bib-0088] Wang, Y. H. , A. Q. Jiao , C. Qiu , et al. 2022. “A Combined Enzymatic and Ionic Cross‐Linking Strategy for Pea Protein/Sodium Alginate Double‐Network Hydrogel With Excellent Mechanical Properties and Freeze‐Thaw Stability.” Food Hydrocolloids 131: 107737. 10.1016/j.foodhyd.2022.107737.

[crf370201-bib-0089] Wang, Y. H. , Q. Liu , Y. Y. Yang , C. Qiu , A. Q. Jiao , and Z. Y. Jin 2023. “Fabrication of a Double‐Network High Internal Phase Emulsion Gel Stabilized by Bacterial Cellulose Nanofibrils: Enhancement of Heat Stability and 3D Printing.” Food Hydrocolloids 143: 108872. 10.1016/j.foodhyd.2023.108872.

[crf370201-bib-0090] Wu, C. , X. Yan , T. Wang , W. Ma , X. Xu , and M. Du 2019. “A Self‐sorted Gel Network Formed by Heating a Mixture of Soy and Cod Proteins.” Food Funct 10, no. 8: 5140–5151. 10.1039/c9fo00560a. 31368476

[crf370201-bib-0091] Xu, Q. Q. , B. K. Qi , L. Han , et al. 2021. “Study on the Gel Properties, Interactions, and pH Stability of Pea Protein Isolate Emulsion Gels as Influenced by Inulin.” Lwt‐Food Science and Technology 137: 110421. 10.1016/j.lwt.2020.110421.

[crf370201-bib-0092] Xu, W. , H. Sun , Y. Jia , et al. 2023. “Pickering Emulsions Synergistic Stabilized with Konjac Glucomannan and Xanthan Gum/Lysozyme Nanoparticles: Structure, Protection and Gastrointestinal Digestion.” Carbohydrate Polymers 305: 120507. 10.1016/j.carbpol.2022.120507. 36737181

[crf370201-bib-0093] Xu, X. , V. V. Jerca , and R. Hoogenboom 2021. “Bioinspired Double Network Hydrogels: From Covalent Double Network Hydrogels via Hybrid Double Network Hydrogels to Physical Double Network Hydrogels.” Mater Horiz 8, no. 4: 1173–1188. 10.1039/d0mh01514h. 34821910

[crf370201-bib-0094] Xu, X. Y. , Y. H. Zhang , M. H. Han , and Q. Guo 2024. “Whey Protein Fibrils Enhance Fat‐Related Texture of Emulsion Systems: Translating Structural Changes to Textural Perception.” Food Hydrocolloids 146: 109208. 10.1016/j.foodhyd.2023.109208.

[crf370201-bib-0095] Yan, J. , X. P. Liang , C. C. Ma , D. J. McClements , X. B. Liu , and F. G. Liu 2021. “Design and Characterization of Double‐Cross‐Linked Emulsion Gels Using Mixed Biopolymers: Zein and Sodium Alginate.” Food Hydrocolloids 113: 106473. 10.1016/j.foodhyd.2020.106473.

[crf370201-bib-0096] Yang, X. , T. Gong , D. Li , A. Li , L. Sun , and Y. Guo 2019. “Preparation of High Viscoelastic Emulsion Gels Based on the Synergistic Gelation Mechanism of xanthan and konjac glucomannan.” Carbohydrate Polymers 226: 115278. 10.1016/j.carbpol.2019.115278. 31582087

[crf370201-bib-0097] Yang, X. , T. Gong , Y. H. Lu , A. Li , L. Sun , and Y. Guo 2020. “Compatibility of Sodium Alginate and Konjac Glucomannan and Their Applications in Fabricating Low‐Fat Mayonnaise‐Like Emulsion Gels.” Carbohydrate Polymers 229: 115468. 10.1016/j.carbpol.2019.115468. 31826449

[crf370201-bib-0098] Yin, Y. , Q. Gu , X. Liu , F. Liu , and D. J. McClements 2023. “Double Network Hydrogels: Design, Fabrication, and Application in Biomedicines and Foods.” Advances in Colloid and Interface Science 320: 102999. 10.1016/j.cis.2023.102999. 37783067

[crf370201-bib-0099] Yiu, C. C. , S. W. Liang , K. Mukhtar , W. Kim , Y. Wang , and C. Selomulya 2023. “Food Emulsion Gels from Plant‐Based Ingredients: Formulation, Processing, and Potential Applications.” Gels 9, no. 5: 366. 10.3390/gels9050366. 37232958 PMC10217108

[crf370201-bib-0100] Zetzl, A. K. , A. G. Marangoni , and S. Barbut 2012. “Mechanical Properties of Ethylcellulose Oleogels and Their Potential for Saturated Fat Reduction in Frankfurters.” Food Funct 3, no. 3: 327–337. 10.1039/c2fo10202a. 22377795

[crf370201-bib-0101] Zhang, H. , A. Wei , S. Zhou , et al. 2024. “Effect of the Substitution of Butter by Double Cross‐Linked Egg Yolk Granules/Sodium Alginate Emulsion Gel on Properties of Baking Dough During Frozen Storage.” Food Chemistry 438: 137965. 10.1016/j.foodchem.2023.137965. 37992605

[crf370201-bib-0102] Zhang, M. , Y. Yang , and N. C. Acevedo 2020a. “Effect of Oil Content and Composition on the Gelling Properties of Egg‐SPI Proteins Stabilized Emulsion Gels.” Food Biophysics 15, no. 4: 473–481. 10.1007/s11483-020-09646-8.

[crf370201-bib-0103] Zhang, M. , Y. Yang , and N. C. Acevedo 2020b. “Effects of Pre‐heating Soybean Protein Isolate and Transglutaminase Treatments on the Properties of Egg‐Soybean Protein Isolate Composite Gels.” Food Chemistry 318: 126421. 10.1016/j.foodchem.2020.126421. 32126461

[crf370201-bib-0104] Zhang, M. , L. Yin , W. Yan , C. Gao , and X. Jia 2022. “Preparation and Characterization of a Novel Soy Protein Isolate‐Sugar Beet Pectin Emulsion Gel and Its Application as a Multi‐Phased Nutrient Carrier.” Foods 11, no. 3: 469. 10.3390/foods11030469. 35159619 PMC8833956

[crf370201-bib-0105] Zhang, Q. , M. Wu , X. Hu , et al. 2019. “A Novel Double‐Network, Self‐Healing Hydrogel Based on Hydrogen Bonding and Hydrophobic Effect.” Macromolecular Chemistry and Physics 221, no. 3: 1900320. 10.1002/macp.201900320.

[crf370201-bib-0106] Zhang, Q. , L. Yin , F. Chen , et al. 2021. “Effect of Soybean Oil Content on Textural, Rheological, and Microstructural Properties of WBAXs‐SPI Emulsion‐Filled Gels.” Journal of Texture Studies 52, no. 2: 251–259. 10.1111/jtxs.12581. 33410521

[crf370201-bib-0107] Zhang, T. , X. Xu , L. Ji , et al. 2017. “Phase Behaviors Involved in Surimi Gel System: Effects of Phase Separation on Gelation of Myofibrillar Protein and Kappa‐Carrageenan.” Food Research International 100, no. Pt 1: 361–368. 10.1016/j.foodres.2017.07.025. 28873698

[crf370201-bib-0108] Zhang, X. , S. Zhang , M. Zhong , B. Qi , and Y. Li 2022. “Soy and Whey Protein Isolate Mixture/Calcium Chloride Thermally Induced Emulsion Gels: Rheological Properties and Digestive Characteristics.” Food Chemistry 380: 132212. 10.1016/j.foodchem.2022.132212. 35139479

[crf370201-bib-0109] Zhao, Y. L. , X. X. Han , N. N. Hu , C. B. Zhao , Y. Z. Wu , and J. S. Liu 2023. “Study on Properties of TGase‐Induced Pea Protein‐Zein Complex Gels.” Journal of Food Engineering 354: 111578. 10.1016/j.jfoodeng.2023.111578.

[crf370201-bib-0110] Zheng, W. , H. Zhang , J. Wang , et al. 2022. “Pickering Emulsion Hydrogel Based on Alginate‐Gellan Gum With Carboxymethyl Chitosan as a pH‐Responsive Controlled Release Delivery System.” International Journal of Biological Macromolecules 216: 850–859. 10.1016/j.ijbiomac.2022.07.223. 35914551

[crf370201-bib-0111] Zhu, F. 2019. “Starch Based Pickering Emulsions: Fabrication, Properties, and Applications.” Trends in Food Science & Technology 85: 129–137. 10.1016/j.tifs.2019.01.012.

